# Impact of skin-to-skin contact on acute procedural pain in newborns: a systematic review and meta-analysis

**DOI:** 10.1016/j.jped.2025.101442

**Published:** 2025-09-11

**Authors:** Lizandro de Andrade Teles, Francisco Plácido Nogueira Arcanjo, Kailane Martins Cardoso, Jeferson de Sousa Justino

**Affiliations:** Universidade Federal do Ceará (UFC), Programa de Pós-Graduação em Ciências da Saúde (PPGCE), Sobral, CE, Brazil

**Keywords:** Neonatal pain, Skin-to-Skin contact, Kangaroo care, Pain Management, Systematic review

## Abstract

**Objective:**

To systematically evaluate the effectiveness of Skin-to-Skin Contact (SSC) in reducing procedural pain in neonates, compared to standard care and other non-pharmacological interventions.

**Data Sources:**

A comprehensive search was conducted in major electronic databases and gray literature up to July 2025. The review followed PRISMA and MECIR guidelines and included randomized controlled trials (RCTs) that used validated neonatal pain scales. Risk of bias was assessed using RoB 2.0, and the certainty of evidence was rated using the GRADE approach.

**Summary of Findings:**

Twenty-nine RCTs involving 2995 neonates were included. SSC significantly reduced procedural pain compared to standard care (SMD = −1.13; 95 % CI: −1.54 to −0.72; *p* < 0.00001), although heterogeneity was high (I² = 93 %). Subgroup analyses suggested that heterogeneity was partially due to differences in pain assessment timing and study design. Compared to other interventions, SSC showed similar efficacy to carbohydrate solutions (SMD = 0.05; 95 % CI: −0.34 to 0.23), superior efficacy to swaddling (SMD = −0.86; 95 % CI: −1.38 to −0.34), and inferior efficacy to breastfeeding (SMD = 0.44; 95 % CI: 0.21 to 0.66).

**Conclusion:**

SSC is an effective intervention for reducing procedural pain in neonates, particularly when compared to standard care and swaddling. However, its equivalence to carbohydrate solutions and inferiority to breastfeeding should be interpreted with caution due to methodological limitations and variability across studies. Further high-quality, large-scale RCTs are needed to improve the certainty of the evidence and guide clinical practice.

## Introduction

Skin-to-skin contact (SSC), also known as Kangaroo Mother Care, is defined as placing a diaper-clad newborn in an upright position directly against the bare chest of the caregiver, ensuring direct skin-to-skin contact. According to the World Health Organization (2003), the infant is typically placed naked except for a diaper and sometimes a cap, to maximize skin contact and thermal regulation.^1^,^2^ This intervention promotes warmth, bonding, and physiological regulation through direct skin contact and multisensory stimulation. SSC is widely endorsed for its role in enhancing neonatal outcomes, particularly in preterm and low-birth-weight infants.^3^, ^4^, ^5^

Emerging evidence suggests that SSC exerts analgesic effects through multiple biological and physiological mechanisms, including the regulation of cortisol and endogenous opioid levels, stabilization of heart rate and respiratory patterns, and modulation of behavioral states that reduce stress.^6^ Additionally, SSC promotes autonomic balance and supports the infant’s immature pain-inhibitory pathways by minimizing sensory overload and encouraging parental presence during painful experiences.^7^ To maximize these analgesic benefits, SSC is generally recommended to be initiated as early as possible, ideally immediately after birth or before a painful procedure, and maintained continuously for a minimum of 15 to 30 min before and during the intervention, as specified by the Brazilian Ministry of Health guidelines.^2^

Neonatal pain, long underestimated in clinical settings, remains a critical ethical and therapeutic concern in Neonatal Intensive Care Units (NICUs). The assumption that neonates, particularly preterm infants, have a diminished capacity to perceive pain has been refuted by neurophysiological and behavioral studies since the 1980s. Research by Anand and Hickey (1987) ^8^ demonstrated that neonates possess the anatomical and functional capacity to perceive nociceptive stimuli, though their immature inhibitory systems may amplify pain responses.^9^

In this context, the accurate assessment of neonatal pain is essential. Although over 40 pain scales have been described in the literature, only a few—such as the Neonatal Infant Pain Scale (NIPS), Neonatal Facial Coding System (NFCS), Premature Infant Pain Profile-Revised (PIPP-R), and the Neonatal Pain, Agitation, and Sedation Scale (N-PASS)—have been validated and widely adopted in clinical practice.^9^ These tools, however, require trained personnel and careful interpretation due to the limited ability of neonates, particularly preterms, to exhibit clear behavioral indicators of pain.^10^

Although SSC has been increasingly integrated into neonatal care, the literature presents significant heterogeneity in study designs, outcome measures, and intervention protocols. Furthermore, most reviews to date have focused primarily on comparisons between SSC and standard care or placebo.^11^,^12^ Few have systematically examined how SSC compares to other non-pharmacological interventions, such as oral sucrose, breastfeeding, or swaddling—strategies commonly used in clinical practice.^11^,^13^,^14^

This knowledge gap limits the ability to tailor evidence-based pain management strategies to specific care settings. This study aims to address that gap by conducting a systematic review and meta-analysis of randomized controlled trials that compare SSC not only to standard care but also to other validated non-pharmacological interventions.

The guiding question for this review is: *In newborns undergoing painful procedures, does skin-to-skin contact reduce pain compared to standard care or other non-pharmacological interventions?* Only studies employing validated pain assessment tools were included.

To enhance methodological transparency, the key elements of the PICO framework for this review are as follows: the Population consists of neonates—both term and preterm—undergoing clinically indicated painful procedures; the Intervention is SSC; the Comparators include standard care as well as other non-pharmacological methods (e.g., oral sucrose, breastfeeding, swaddling); and the Outcome is pain, measured using validated neonatal pain assessment scales.

## Methods

This systematic review was conducted in accordance with the Methodological Expectations of Cochrane Intervention Reviews (MECIR) and the PRISMA 2020 guidelines^15^ with a protocol registered in PROSPERO (CRD42024465002).

### Inclusion criteria

Randomized clinical trials assessing pain intensity in newborns of any gestational age using validated pain scales were included. There were no restrictions regarding language, publication status, or date.

### Primary outcome of interest

The primary outcome was the intensity of neonatal pain, measured using validated behavioral and physiological pain assessment tools, including the PIPP, NIPS, NFCS, NPASS, and PICS scales.

### Search strategy

The searches were conducted up to July 2025, across multiple databases, including PubMed, EMBASE, LILACS, SCOPUS, Web of Science, and the Cochrane Library. Additional sources included gray literature (Google Scholar, ProQuest) and clinical trial registries (ClinicalTrials.gov). The reference lists of included studies were also screened for relevant articles. Detailed search strategies are provided in Appendix 1.

### Study selection and data extraction

Two independent reviewers conducted the screening in two phases (title/abstract and full text) and extracted data using standardized spreadsheets. Discrepancies were resolved by consensus. The extracted variables included methodological characteristics, sample details, interventions, outcomes, and main conclusions.

### Assessment of risk of bias and certainty of the evidence

Risk of bias was assessed using the RoB 2.0 tool, which considers five domains. The certainty of the evidence was summarized in the summary of findings tables according to the GRADE approach.^16^,^17^

### Measures of effect size

For continuous outcomes measured using different pain scales across studies, the standardized mean difference (SMD) with 95 % confidence intervals (CIs) was calculated. The use of SMD allows for the combination of results from studies employing different measurement instruments by standardizing effect sizes on a uniform scale, facilitating meaningful meta-analysis across heterogeneous outcomes.^16^

### Units of analysis and methods of data synthesis

The unit of analysis was the individual. In studies with multiple arms or multiple time points, the time point closest to the pain-inducing stimulus was used for analysis.

### Heterogeneity and publication bias

Heterogeneity was assessed using Cochran’s Q test and the I^2^ statistic, with interpretation based on the Cochrane Handbook guidelines. Publication bias was analyzed when ten or more studies were available per comparison.^16^

### Data synthesis and additional analyses

Meta-analyses were conducted using a random-effects model with the inverse variance method in R (meta package) and Revman 5.4.1. In the presence of substantial heterogeneity, subgroup analyses were performed based on gestational age, pain type, measurement scale, and pain assessment timing. Sensitivity analyses were conducted by excluding studies with a high risk of bias.

## Results

### Identification of studies

**Figure 1:**
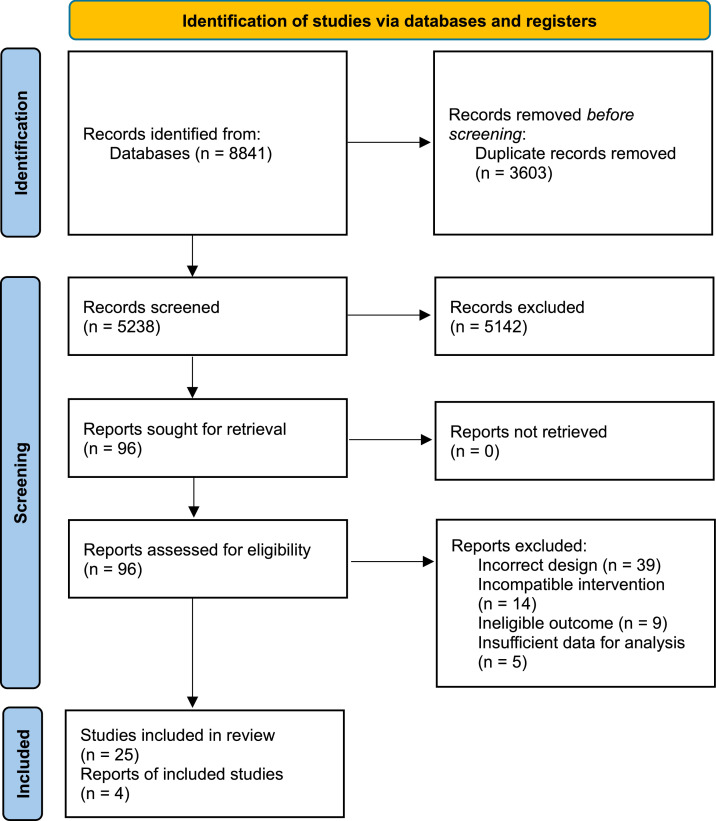
Flowchart of the study selection process.

The search in the databases retrieved 8841 records. After removing duplicates, the titles and abstracts of 5238 references were reviewed. A total of 5142 references were excluded for not meeting the eligibility criteria for this systematic review. Therefore, a full-text review was conducted on ninety-six of the selected references. After the full-text review, sixty-seven studies were excluded for reasons described in Figure 1. As a result, a total of twenty-nine studies were included, of which twenty-five were full-text articles and four were reports, as illustrated in Figure 1 below.

All included studies had an appropriate methodological design. The interventions analyzed involved SSC or the kangaroo method as the main intervention, with pain assessment using validated scales. The studies covered neonatal populations, both term and preterm, in different painful procedures. The characteristics of the studies are presented in Supplementary Table 1.

### Risk of bias in the included studies

**Figure 2:**
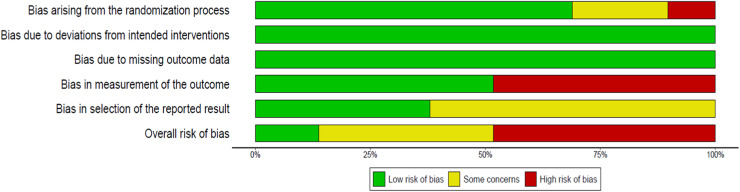
Overall risk of bias (RoB 2) summary: assessment of skin-to-skin contact for procedural pain in newborns.

The risk of bias assessment for the 29 studies included in the review,^18^, ^19^, ^20^, ^21^, ^22^, ^23^, ^24^, ^25^, ^26^, ^27^, ^28^, ^29^, ^30^, ^31^, ^32^, ^33^, ^34^, ^35^, ^36^, ^37^, ^38^, ^39^, ^40^, ^41^, ^42^, ^43^, ^44^, ^45^, ^46^ conducted using the RoB 2 tool, revealed significant methodological variations across the analyzed domains. In the randomization domain, 69 % of the studies were considered low risk,^18^, ^19^, ^20^^,^^22^,^24^,^26^^,^^28^, ^29^, ^30^^,^^32^^,^^34^, ^35^, ^36^^,^^38^^,^^40^, ^41^, ^42^, ^43^, ^44^^,^^46^ 20.7 % raised some concerns, ^23^,^25^,^31^,^33^,^39^,^45^ and 10.3 % were classified as high risk.^21^,^27^,^37^ Adherence to the protocol and the absence of outcome losses were consistent, with all studies classified as low risk in these domains.^18^, ^19^, ^20^, ^21^, ^22^, ^23^, ^24^, ^25^, ^26^, ^27^, ^28^, ^29^, ^30^, ^31^, ^32^, ^33^, ^34^, ^35^, ^36^, ^37^, ^38^, ^39^, ^40^, ^41^, ^42^, ^43^, ^44^, ^45^, ^46^ On the other hand, outcome measurement demonstrated methodological weaknesses, with only 51.7 % of studies classified as low risk 18, 19, 20^,^^24^,^25^,^28^,^29^,^34^, ^35^, ^36^^,^^38^^,^^43^, ^44^, ^45^, ^46^ and 48.3 % as high risk.^21^,^23^,^24^,^26^,^27^^,^^30^, ^31^, ^32^, ^33^^,^^37^^,^^39^, ^40^, ^41^, ^42^ The selection of reported outcomes also showed limitations, with 62.1 % of studies raising ‘some concerns’.^18^, ^19^, ^20^^,^^23^,^24^,^29^,^31^^,^^33^, ^34^, ^35^^,^^37^, ^38^, ^39^^,^^41^,^42^^,^^44^, ^45^, ^46^ As a result, the overall risk of bias was classified as high in 48.3 % of studies,^21^,^23^,^24^,^26^,^27^^,^^30^, ^31^, ^32^, ^33^^,^^37^^,^^39^, ^40^, ^41^, ^42^ with only 13.8 % considered low risk (Figure 2).^22^,^28^,^36^,^43^

In the analysis by the comparator:

• SSC vs. Control (*n* = 19): 63.2 % of the studies had low risk in randomization,^18^, ^19^, ^20^^,^^22^,^24^^,^^28^, ^29^, ^30^^,^^32^,^35^,^36^,^44^ but 42.1 % were at high risk for outcome measurement.^21^,^23^,^24^ ^30^,^32^,^33^,^37^,^39^ Only 15.8 % of the studies were considered low risk overall (Supplementary Figure 1).^22^,^28^,^36^
• SSC vs. Carbohydrate Solution (*n* = 9): While randomization was adequate in 77.8 % of the studies,^22^,^30^,^34^,^42^,^43^,^46^ more than half (56.6 %) had high risk in outcome measurement,^21^,^27^,^30^,^41^ resulting in 56.6 % of studies showing high overall bias (Supplementary Figure 2).^21^,^27^,^30^,^41^,^42^
• SSC vs. Breastfeeding (*n* = 4): Three studies were classified as having high overall risk (75 %),^21^,^26^,^31^ with the greatest weakness in outcome measurement (Supplementary Figure 3).
• SSC vs. Swaddling (*n* = 5): Despite proper randomization and no losses to follow-up, 60 % had high risk in outcome measurement,^24^,^26^,^40^ with 60 % of studies showing high overall bias (Supplementary Figure 4).^24^,^26^,^40^

The high proportion of studies with a high risk of bias may be attributed to intrinsic characteristics of the intervention under investigation. SSC between mother and infant following birth, when compared to other forms of neonatal care, poses significant methodological challenges, particularly with respect to the blinding of participants and outcome assessors. Although blinding is technically feasible in certain contexts, many authors acknowledge this limitation, given that the intervention is visible and involves direct physical interaction, thereby complicating the implementation of effective masking strategies. Additionally, ethical and operational constraints often preclude complete blinding, which may have adversely impacted the domains of outcome measurement and outcome selection, contributing to the overall risk of bias observed across the studies.

### Effect of the interventions

#### Skin-to-Skin Contact Versus Control for Procedural Pain in Newborns

**Figure 3:**
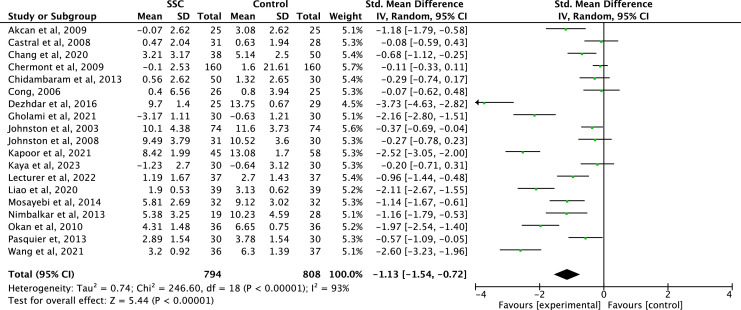
Meta-analysis: skin-to-skin contact versus control for procedural pain in newborns.

The meta-analysis of 19 studies (*n* = 1602) showed a significant reduction in neonatal pain with SSC compared to control (SMD = −1.13; 95 % CI: −1.54 to −0.72; *p* < 0.00001). Heterogeneity was high (I^2^ = 93 %), reflecting substantial variability across studies. Among the included trials, Dezhdar et al.^25^ (SMD = −3.73), Wang et al.^44^ (SMD = −2.60), Kapoor et al.^30^ (SMD = −2.52), and Liao et al.^33^ (SMD = −2.11) reported the largest analgesic effects. These pronounced differences underscore the relevance of conducting subgroup analyses to identify clinical or methodological factors influencing treatment outcomes (Figure 3).

#### Subgroup Analysis of Skin-to-Skin Contact versus Control for Procedural Pain in Newborns

The analysis by gestational age did not identify statistically significant differences, indicating that the analgesic effect of SSC was consistent between term and preterm newborns (Supplementary Figure 5).

Regarding the type of pain, SSC was significantly effective across different procedures, although the magnitude of the effect varied according to the stimulus (Supplementary Figure 6).

The type of pain assessment scale did not significantly influence the results, suggesting that the analgesic effect of SSC was robust regardless of the instrument used (Supplementary Figure 7).

In contrast, the timing of pain assessment influenced the observed effect, with more pronounced analgesic responses when pain was evaluated during or immediately after the procedure (Supplementary Figure 8).

#### Skin-to-Skin Contact versus Carbohydrate Solution for Procedural Pain in Newborns

**Figure 4:**
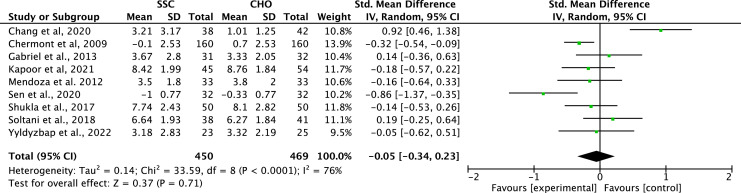
Meta-analysis skin-to-skin contact versus carbohydrate solution for procedural pain in newborns.

The meta-analysis of nine randomized controlled trials (*n* = 919) that compared SSC with carbohydrate solution (CHO) showed no statistically significant difference between interventions regarding neonatal pain reduction (SMD = 0.05; 95 % CI: 0.34 to 0.23; *p* = 0.71). The heterogeneity was considered substantial (I2 = 76 %), indicating relevant variability among the included studies (Figure 4).

#### Subgroup Analysis of Skin-to-Skin Contact versus Carbohydrate Solution for Procedural Pain in Newborns

The analysis by gestational age showed no statistically significant differences between term and preterm newborns, although there was a trend toward greater benefit from SSC in preterm infants (Supplementary Figure 9).

In the analysis by type of pain, no significant differences were observed between SSC and CHO for heel lance or venipuncture. A superior effect of SSC was identified only in vaccination procedures (Supplementary Figure 10).

The analysis by pain assessment scale revealed relevant variations. Only the study using the NPASS scale demonstrated a significant benefit of CHO. The interaction test was statistically significant, suggesting that the type of scale may influence the estimated effect size (Supplementary Figure 11).

Regarding the timing of pain assessment, SSC was effective only at 5 min post-procedure, with no consistent differences observed at other time points (Supplementary Figure 12).

#### Skin-to-Skin Contact versus Breastfeeding for Procedural Pain in Newborns

**Figure 5:**
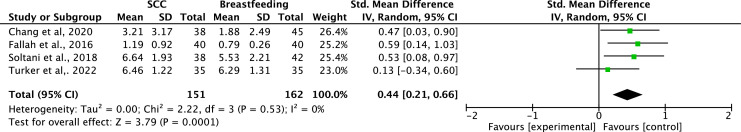
Meta-analysis: skin-to-skin contact versus breastfeeding for procedural pain in newborns.

The meta-analysis comparing SSC with breastfeeding included four studies (*n* = 313), showing a significant effect favoring breastfeeding (SMD = 0.44; 95 % CI: 0.21 to 0.66; *p* = 0.0001). Heterogeneity was absent (I² = 0 %), indicating high consistency across studies in terms of effect size and direction. Given the lack of variability, subgroup analyses were not necessary, as the findings appear robust and unaffected by potential effect modifiers (Figure 5).

#### Skin-to-skin contact versus Swaddling for Procedural Pain in Newborns

**Figure 6:**
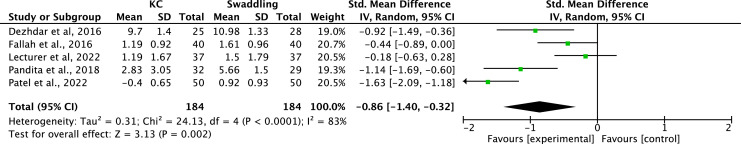
Meta-analysis: skin-to-skin contact versus swaddling for procedural pain in newborns.

The meta-analysis comparing SSC and Swaddling included five studies with 184 neonates per group. The overall effect significantly favored SSC (SMD = −0.86; 95 % CI: −1.38 to −0.34; *p* = 0.0011), indicating superior pain reduction. Despite substantial heterogeneity (I² = 83.4 %), the direction of effect was consistently favorable to SSC, notably in studies by Patel and Pandita ^38^,^40^ (Figure 6).

#### Subgroup Analysis of Skin-to-Skin Contact versus Swaddling for Procedural Pain in Newborns.

In the analysis by gestational age, SSC significantly reduced pain in full-term newborns but not in preterm infants. There was no significant subgroup interaction (Supplementary Figure 13).

Regarding the type of pain, SSC was more effective than swaddling for pain associated with vaccination, venipuncture, and vitamin administration. No significant difference was observed for procedures involving peripheral vascular access. The magnitude of the effect varied according to the type of painful stimulus (Supplementary Figure 14).

The analysis by pain assessment scale showed consistent analgesic effects of SSC when measured with both the NIPS and PIPP scales. While heterogeneity was high in studies using NIPS and absent in those using PIPP, no statistically significant difference was detected between subgroups, suggesting robustness of the intervention regardless of the instrument applied (Supplementary Figure 15).

In relation to the timing of pain assessment, a greater analgesic effect was observed when pain was measured 5 min after the procedure. Effects were less pronounced or non-significant at earlier time points, indicating that timing significantly influenced the estimated effectiveness of SSC (Supplementary Figure 16).

#### Skin-to-Skin Contact versus Non-Nutritive Sucking for Procedural Pain in Newborns

**Figure 7:**
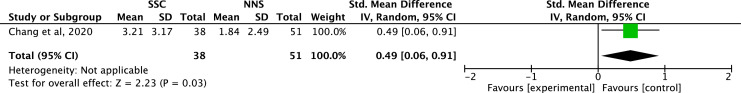
Meta-analysis: KC versus non-nutrictive sucking for procedural pain in newborns.

Although planned in the protocol, the meta-analysis comparing SSC with Non-Nutritive Sucking (NNS) could not be conducted due to the inclusion of only one eligible study. This trial favored non-nutritive sucking, but the absence of additional randomized data precluded quantitative synthesis (Figure 7).

### Publication bias analysis

**Figure 8:**
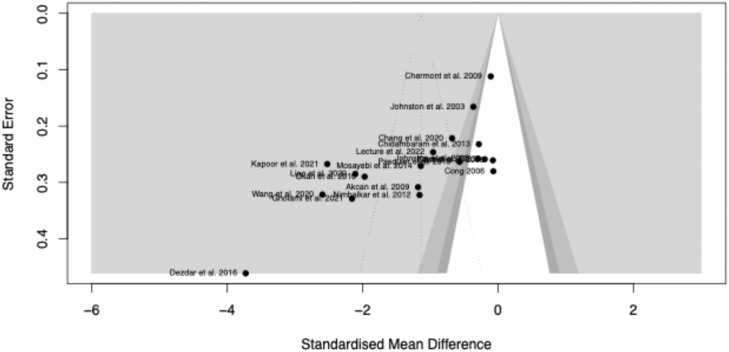
Funnel plot skin-to-skin contact versus control for procedural pain in newborns.

For the comparison between SSC and control, potential publication bias was evaluated using funnel plot asymmetry, Egger’s test, and a trim-and-fill analysis. Visual inspection of the funnel plot suggested asymmetry, indicating underreporting of studies unfavorable to KC. Egger’s test confirmed significant asymmetry (*t* = −3.99; *p* = 0.0009), suggesting overestimation of effect sizes in smaller studies (Figure 8).

The Duval and Tweedie trim-and-fill method imputed six potentially missing studies, resulting in an adjusted effect size of SMD = −0.48 (95 % CI: −1.09 to 0.13; *p* = 0.12), a non-significant result, contrasting with the original pooled estimate. Heterogeneity remained high (I^2^ = 95.6 %), reinforcing the need for cautious interpretation.

For other comparisons, bias assessments were not conducted due to the inclusion of fewer than ten studies per outcome, consistent with methodological recommendations to avoid low-powered analyses.

### Summary of findings

SSC significantly reduced procedural pain in newborns compared to standard care, though the evidence is of low certainty. Its efficacy was comparable to CHOs (very low certainty), inferior to breastfeeding (moderate certainty), and superior to swaddling (moderate certainty).

A detailed *Summary of Findings* table, including effect estimates, number of participants, and GRADE ratings, is provided in the Supplementary Material.

## Discussion

This review extends previous work, such as Johnston et al. (2014),^47^ by incorporating comprehensive subgroup analyses that examine the efficacy of SSC according to different types of painful stimuli, gestational age groups, pain assessment scales, and timing of pain measurement. These detailed analyses provide a more nuanced understanding of SSC’s analgesic effects across varied clinical contexts, enhancing the practical applicability of the findings. By directly comparing SSC with multiple non-pharmacological interventions, this study offers critical insights to inform tailored pain management strategies in neonatal care.

This systematic review and meta-analysis included twenty-nine randomized controlled trials that evaluated the efficacy of SSC in pain control during painful clinical procedures in neonates. Compared to routine care or absence of intervention, SSC demonstrated significant efficacy in reducing neonatal pain (SMD = 1.13; 95 % CI: 1.54 to 0.72; *p* < 0.00001). While SMDs are a statistical approach to standardize effects across different pain scales, their clinical interpretation can be informed by established thresholds: values around 0.2 are considered small, 0.5 moderate, and 0.8 large, according to Cohen’s benchmarks.^16^ Thus, the observed SMD in the review represents a large and clinically meaningful effect size. It should be noted, however, that no universal minimal clinically important difference (MCID) has been formally defined for neonatal pain scales.

There was high heterogeneity among the studies (I² = 93 %), indicating considerable variability of results. The analysis of subgroups by gestational age did not identify statistically significant differences; SSC showed consistent favorable effects in term and premature newborns. Additional analyses by type of pain, scale used, and time of measurement suggest that SSC is most effective during or immediately after the painful procedure.

While SSC and CHOs demonstrated similar effectiveness (SMD = 0.05; 95 % CI: 0.34 to −0.23; *p* = 0.71), breastfeeding was superior to SSC (SMD = 0.44; 95 % CI: 0.21 to 0.66; *p* = 0.0001), and SSC was significantly more effective than swaddling (SMD = 0.86; 95 % CI: 1.38 to 0.34; *p* = 0.0011). These head-to-head comparisons provide valuable insight into the relative efficacy of common non-pharmacological pain interventions in neonatal care, a dimension rarely addressed in previous reviews.

## Limitations

This review has several limitations that should be considered when interpreting the findings.

*-Study Design and Risk of Bias:* Although all included studies were randomized controlled trials, many presented a high risk of bias, primarily due to the inherent challenges in blinding skin-to-skin contact interventions. This issue particularly affected the measurement of outcomes.

*-Participants and Sample Size:* The included studies involved both term and preterm neonates. However, most had small sample sizes (fewer than 50 participants per group), limiting statistical power and reducing the precision of effect estimates.

*-Intervention Protocols and Comparisons:* There was significant heterogeneity in the application of interventions and control conditions. While SSC was compared to various non-pharmacological strategies (e.g., sucrose, breastfeeding, swaddling), inconsistencies in how these were delivered across studies contributed to variability in the results.

*-Pain Assessment Tools:* Although all studies used validated neonatal pain scales, the diversity of instruments (e.g., PIPP, NIPS, NFCS, N-PASS) introduced inconsistency and hindered direct comparability.

*Certainty of Evidence:* Using the GRADE approach, the certainty of evidence ranged from moderate to very low. This was due to a combination of high risk of bias, small sample sizes, wide confidence intervals, and substantial heterogeneity. Publication bias was also evident.

*Review Process:* Despite adherence to rigorous methodological standards (MECIR and PRISMA) and an extensive search strategy, the possibility of missing relevant studies, particularly from grey literature, cannot be excluded.

Agreements and disagreements with other studies or reviews

The present results largely agree with previous reviews, such as that of Johnston et al. (2014),^47^ confirming the overall effectiveness of SSC in relieving neonatal pain. However, this review expanded the scope to include additional comparisons (CHOs, Breastfeeding and Swaddling) and detailed analyses by subgroups, highlighting important differences in the relative efficacy of SSC against other non-pharmacological interventions, especially compared to breastfeeding.

### Practical implications

The findings of this review support the integration of SSC as a standard non-pharmacological strategy for procedural pain management in various clinical settings. In Neonatal Intensive Care Units (NICUs), SSC can be implemented during routine procedures such as heel lancing, venipuncture, or vaccinations, offering a simple, low-cost, and humanizing approach that requires minimal equipment and training. In delivery rooms, SSC immediately after birth not only promotes bonding and physiological stabilization but also provides an effective method to alleviate pain associated with early interventions like vitamin K administration or blood sampling. The widespread applicability of SSC across these settings highlights its potential to improve neonatal outcomes and enhance the quality of care in both high- and low-resource environments.

## Conclusions

SSC is effective in reducing procedural pain in neonates, particularly when compared to standard care and swaddling. However, its analgesic effect appears comparable to that of carbohydrate solutions and inferior to breastfeeding. These findings suggest that SSC should be considered a core component of non-pharmacological pain management, especially in settings where breastfeeding is not immediately feasible. Given its simplicity, safety, and accessibility, SSC can be effectively implemented in both NICUs and outpatient settings. In clinical practice, combining SSC with other strategies—such as sucrose administration or non-nutritive sucking—may offer enhanced analgesic benefits. For instance, SSC combined with oral sucrose could be particularly valuable during short procedures like heel sticks or venipuncture in preterm infants, while SSC with breastfeeding may be ideal during vaccinations or blood draws in full-term neonates. To support broader implementation, clinical protocols should adapt SSC-based pain management according to available resources, type of procedure, and infant maturity. Future high-quality trials are needed to validate these combinations and optimize their application across diverse care environments. A practical decision framework or summary table differentiating recommendations by clinical setting (e.g., NICU vs. outpatient) may further enhance bedside usability.

## Conflicts of interest

The authors declare no conflicts of interest.

## Appendix Supplementary materials

Image, application 1

Image, application 2

Image, application 3

Image, application 4

## Footnotes

Supplementary material associated with this article can be found in the online version at doi:10.1016/j.jped.2025.101442.
