# Supplementary material for: Impact of skin-to-skin contact on acute procedural pain in newborns: a systematic review and meta-analysis
Source: J Pediatr (Rio J). 2025 Sep 11;101(6):101442. doi: 10.1016/j.jped.2025.101442 (PMC12744622; doi:10.1016/j.jped.2025.101442)
Supplement: Supplementary file 1 [file mmc1.docx]

**JPED-D-25-00185_Supplementary Material_Appendix**

**Appendix 1** - Databases and search strategies.

| Database | Search  strategy | Results  Feb 01^st^ 2024 |
| --- | --- | --- |
| Medline / PubMed | ("infant, newborn"[MeSH Major Topic:noexp] OR "Newborn Infant"[Title/Abstract] OR "Newborn Infants"[Title/Abstract] OR "Newborns"[Title/Abstract] OR "Newborn"[Title/Abstract] OR "Neonate"[Title/Abstract] OR "Neonates"[Title/Abstract] OR "baby"[Title/Abstract] OR "babies"[Title/Abstract] OR "infant, premature"[MeSH Terms] OR "Premature Infant"[All Fields] OR "Preterm Infants"[All Fields] OR "Preterm Infant"[All Fields] OR "Premature Infants"[All Fields] OR "Neonatal Prematurity"[All Fields] OR "premature"[Title/Abstract] OR "preterm"[Title/Abstract] OR "prematurity"[Title/Abstract] OR "infant, low birth weight"[MeSH Terms] OR "low birth weight infant"[All Fields] OR "low birth weight infant"[All Fields] OR "Low-Birth-Weight Infants"[All Fields] OR "Low Birth Weight"[All Fields] OR "Low Birth Weights"[All Fields] OR "infant, very low birth weight"[MeSH Terms] OR "very low birth weight infant"[All Fields] OR "very low birth weight infant"[All Fields] OR "Very-Low-Birth-Weight Infants"[All Fields] OR "Very Low Birth Weight"[All Fields] OR "infant, extremely low birth weight"[MeSH Terms] OR "Extremely Low Birth Weight Infant"[All Fields] OR "infant, small for gestational age"[MeSH Terms] OR "Infant Small for Gestational Age"[All Fields] OR "Small for Gestational Age Infant"[All Fields]) AND ("pain, procedural"[MeSH Terms] OR "Procedural Pain"[All Fields] OR "Acute Pain"[MeSH Terms] OR "Acute Pain"[Title/Abstract] OR "Acute Pains"[All Fields] OR "Pain"[MeSH Major Topic:noexp] OR "Pain"[Title/Abstract] OR "Pains"[Title/Abstract] OR "Ache"[Title/Abstract] OR "Aches"[Title/Abstract] OR "Physical Suffering"[All Fields] OR "Physical Sufferings"[All Fields]) AND ("Pain Management"[MeSH Terms] OR "Pain Management"[Title/Abstract] OR "Pain Managements"[All Fields] OR "Pain Measurement"[MeSH Terms] OR "Pain Measurement"[All Fields] OR "Pain Measurements"[All Fields] OR "Pain Assessment"[All Fields] OR "Pain Assessments"[All Fields] OR "Pain Intensity"[All Fields] OR "Pain Intensities"[All Fields] OR "Pain Severity"[All Fields] OR "Pain Severities"[All Fields] OR "Analgesia Tests"[All Fields] OR "Analgesia Test"[All Fields] OR "Pain Scale"[All Fields] OR "Pain Scales"[All Fields] OR "Pain Test"[All Fields] OR "Pain Tests"[All Fields] OR "Nociception Tests"[All Fields] OR "Nociception Test"[All Fields] OR "Formalin Test"[All Fields] OR "Formalin Tests"[All Fields]) AND ("therapeutics"[MeSH Terms] OR "therapeutics"[Title/Abstract] OR "treatment"[Title/Abstract] OR "treatments"[Title/Abstract] OR "therapies"[Title/Abstract] OR "therapy"[Title/Abstract] OR ("kangaroo mother care method"[MeSH Terms] OR "kangaroo mother care method"[All Fields] OR "kangaroo mother care method"[All Fields] OR "Kangaroo Mother Care Methods"[All Fields] OR "kangaroo mother care"[All Fields] OR "kangaroo mother care"[All Fields] OR "kangaroo mother method"[All Fields] OR "kangaroo care"[All Fields] OR "skin to skin care"[All Fields] OR "skin-to-skin"[All Fields] OR "skin-to-skin"[All Fields] OR "skin to skin contact"[All Fields])) | 1,638 |
| Embase | ('newborn'/mj OR 'newborn infant':ti,ab,kw OR 'newborn infants':ti,ab,kw OR 'newborns':ti,ab,kw OR 'newborn':ti,ab,kw OR 'neonate':ti,ab,kw OR 'neonates':ti,ab,kw OR 'baby':ti,ab,kw OR 'babies':ti,ab,kw OR 'prematurity'/exp OR 'premature infant' OR 'preterm infants' OR 'preterm infant' OR 'premature infants' OR 'neonatal prematurity' OR 'premature':ti,ab,kw OR 'preterm':ti,ab,kw OR 'prematurity':ti,ab,kw OR 'low birth weight'/exp OR 'low birth weight infant' OR 'low birth weight infant' OR 'low-birth-weight infants' OR 'low birth weight' OR 'low birth weights' OR 'very low birth weight'/exp OR 'very low birth weight infant' OR 'very low birth weight infant' OR 'very-low-birth-weight infants' OR 'very low birth weight' OR 'extremely low birth weight'/exp OR 'extremely low birth weight infant' OR 'small for gestational age'/exp OR 'infant small for gestational age' OR 'small for gestational age infant') AND ('procedural pain'/exp OR 'procedural pain' OR 'acute pain':ti,ab,kw OR 'acute pains' OR 'pain'/mj OR 'pain':ti,ab,kw OR 'pains':ti,ab,kw OR 'ache':ti,ab,kw OR 'aches':ti,ab,kw OR 'physical suffering' OR 'physical sufferings') AND ('analgesia'/exp OR 'pain management':ti,ab,kw OR 'pain managements' OR 'pain measurement'/exp OR 'pain measurement' OR 'pain measurements' OR 'pain assessment' OR 'pain assessments' OR 'pain intensity' OR 'pain intensities' OR 'pain severity' OR 'pain severities' OR 'analgesia tests' OR 'analgesia test' OR 'pain scale' OR 'pain scales' OR 'pain test' OR 'pain tests' OR 'nociception tests' OR 'nociception test' OR 'formalin test' OR 'formalin tests') AND ('therapy'/exp OR 'therapeutics':ti,ab,kw OR 'treatment':ti,ab,kw OR 'treatments':ti,ab,kw OR 'therapies':ti,ab,kw OR 'therapy':ti,ab,kw OR 'kangaroo care'/exp OR 'kangaroo mother care method' OR 'kangaroo mother care method' OR 'kangaroo mother care methods' OR 'kangaroo mother care' OR 'kangaroo mother care' OR 'kangaroo mother method' OR 'kangaroo care' OR 'skin to skin care' OR 'skin-to-skin' OR 'skin-to-skin' OR 'skin to skin contact') | 2,892 |
| Scopus | TITLE-ABS-KEY("Newborn Infant" OR "Newborn Infants" OR Newborns OR Newborn OR Neonate OR Neonates OR baby OR babies OR "Premature Infant" OR "Preterm Infants" OR "Preterm Infant" OR "Premature Infants" OR "Neonatal Prematurity" OR premature OR preterm OR prematurity OR "Low Birth Weight Infant" OR "Low-Birth-Weight Infant" OR "Low-Birth-Weight Infants" OR "Low Birth Weight" OR "Low Birth Weights" OR "Very Low Birth Weight Infant" OR "Very-Low-Birth-Weight Infant" OR "Very-Low-Birth-Weight Infants" OR "Very Low Birth Weight" OR "Extremely Low Birth Weight Infant" OR "Infant Small for Gestational Age" OR "Small for Gestational Age Infant") AND TITLE-ABS-KEY("Procedural Pain" OR "Acute Pain" OR "Acute Pains" OR Pain OR Pains OR Ache OR Aches OR "Physical Suffering" OR "Physical Sufferings") AND TITLE-ABS-KEY("Pain Management" OR "Pain Managements" OR "Pain Measurement" OR "Pain Measurements" OR "Pain Assessment" OR "Pain Assessments" OR "Pain Intensity" OR "Pain Intensities" OR "Pain Severity" OR "Pain Severities" OR "Analgesia Tests" OR "Analgesia Test" OR "Pain Scale" OR "Pain Scales" OR "Pain Test" OR "Pain Tests" OR "Nociception Tests" OR "Nociception Test" OR "Formalin Test" OR "Formalin Tests") AND TITLE-ABS-KEY(therapeutics OR treatment OR treatments OR therapies OR therapy OR "Kangaroo-Mother Care Method" OR "Kangaroo Mother Care Method" OR "Kangaroo Mother Care Methods" OR "Kangaroo Mother Care" OR "Kangaroo-Mother Care" OR "kangaroo mother method" OR "kangaroo care" OR "skin to skin care" OR "skin-to-skin" OR "skin to skin" OR "skin to skin contact") | 2,321 |
| Web of Science | TS=("Newborn Infant" OR "Newborn Infants" OR Newborns OR Newborn OR Neonate OR Neonates OR baby OR babies OR "Premature Infant" OR "Preterm Infants" OR "Preterm Infant" OR "Premature Infants" OR "Neonatal Prematurity" OR premature OR preterm OR prematurity OR "Low Birth Weight Infant" OR "Low-Birth-Weight Infant" OR "Low-Birth-Weight Infants" OR "Low Birth Weight" OR "Low Birth Weights" OR "Very Low Birth Weight Infant" OR "Very-Low-Birth-Weight Infant" OR "Very-Low-Birth-Weight Infants" OR "Very Low Birth Weight" OR "Extremely Low Birth Weight Infant" OR "Infant Small for Gestational Age" OR "Small for Gestational Age Infant") AND TS=("Procedural Pain" OR "Acute Pain" OR "Acute Pains" OR Pain OR Pains OR Ache OR Aches OR "Physical Suffering" OR "Physical Sufferings") AND TS=("Pain Management" OR "Pain Managements" OR "Pain Measurement" OR "Pain Measurements" OR "Pain Assessment" OR "Pain Assessments" OR "Pain Intensity" OR "Pain Intensities" OR "Pain Severity" OR "Pain Severities" OR "Analgesia Tests" OR "Analgesia Test" OR "Pain Scale" OR "Pain Scales" OR "Pain Test" OR "Pain Tests" OR "Nociception Tests" OR "Nociception Test" OR "Formalin Test" OR "Formalin Tests") AND TS=(therapeutics OR treatment OR treatments OR therapies OR therapy OR "Kangaroo-Mother Care Method" OR "Kangaroo Mother Care Method" OR "Kangaroo Mother Care Methods" OR "Kangaroo Mother Care" OR "Kangaroo-Mother Care" OR "kangaroo mother method" OR "kangaroo care" OR "skin to skin care" OR "skin-to-skin" OR "skin to skin" OR "skin to skin contact") | 590 |
| Cochrane Library | ("Newborn Infant" OR "Newborn Infants" OR Newborns OR Newborn OR Neonate OR Neonates OR baby OR babies OR "Premature Infant" OR "Preterm Infants" OR "Preterm Infant" OR "Premature Infants" OR "Neonatal Prematurity" OR premature OR preterm OR prematurity OR "Low Birth Weight Infant" OR "Low-Birth-Weight Infant" OR "Low-Birth-Weight Infants" OR "Low Birth Weight" OR "Low Birth Weights" OR "Very Low Birth Weight Infant" OR "Very-Low-Birth-Weight Infant" OR "Very-Low-Birth-Weight Infants" OR "Very Low Birth Weight" OR "Extremely Low Birth Weight Infant" OR "Infant Small for Gestational Age" OR "Small for Gestational Age Infant"):ti,ab,kw AND ("Procedural Pain" OR "Acute Pain" OR "Acute Pains" OR Pain OR Pains OR Ache OR Aches OR "Physical Suffering" OR "Physical Sufferings"):ti,ab,kw AND ("Pain Management" OR "Pain Managements" OR "Pain Measurement" OR "Pain Measurements" OR "Pain Assessment" OR "Pain Assessments" OR "Pain Intensity" OR "Pain Intensities" OR "Pain Severity" OR "Pain Severities" OR "Analgesia Tests" OR "Analgesia Test" OR "Pain Scale" OR "Pain Scales" OR "Pain Test" OR "Pain Tests" OR "Nociception Tests" OR "Nociception Test" OR "Formalin Test" OR "Formalin Tests"):ti,ab,kw AND (therapeutics OR treatment OR treatments OR therapies OR therapy OR "Kangaroo-Mother Care Method" OR "Kangaroo Mother Care Method" OR "Kangaroo Mother Care Methods" OR "Kangaroo Mother Care" OR "Kangaroo-Mother Care" OR "kangaroo mother method" OR "kangaroo care" OR "skin to skin care" OR "skin-to-skin" OR "skin to skin" OR "skin to skin contact"):ti,ab,kw | 1003 |
| LILACS | ("Newborn Infant" OR "Newborn Infants" OR newborns OR newborn OR neonate OR neonates OR baby OR babies OR "Premature Infant" OR "Preterm Infants" OR "Preterm Infant" OR "Premature Infants" OR "Neonatal Prematurity" OR premature OR preterm OR prematurity OR "Low Birth Weight Infant" OR "Low-Birth-Weight Infant" OR "Low-Birth-Weight Infants" OR "Low Birth Weight" OR "Low Birth Weights" OR "Very Low Birth Weight Infant" OR "Very-Low-Birth-Weight Infant" OR "Very-Low-Birth-Weight Infants" OR "Very Low Birth Weight" OR "Extremely Low Birth Weight Infant" OR "Infant Small for Gestational Age" OR "Small for Gestational Age Infant" OR "Recém-Nascido" OR "Recém-Nascidos" OR "Criança Recém-Nascida" OR "Crianças Recém-Nascidas" OR "Lactente Recém-Nascido" OR "Lactentes Recém-Nascidos" OR neonato OR neonatos OR "Recién Nacido" OR "Lactante Recién Nacido" OR "Lactantes Recién Nacidos" OR "Niño Recién Nacido" OR "Niños Recién Nacidos" OR "Recién Nacidos" OR "Recém-Nascido Prematuro" OR "Bebe Prematuro" OR "Bebes Prematuros" OR "Lactente Nascido Prematuramente" OR "Lactente Nascido Pré-Termo" OR "Lactente Prematuro" OR "Lactente Pré-Termo" OR "Lactentes Nascidos Prematuramente" OR "Lactentes Nascidos Prematuros" OR "Lactentes Nascidos Pré-Termo" OR "Lactentes Prematuros" OR "Lactentes Pré-Termo" OR "Neonato Prematuro" OR "Neonato Pré-Termo" OR "Neonatos Prematuros" OR "Neonatos Pré-Termo" OR prematuridade OR "Prematuridade Neonatal" OR prematuro OR prematuros OR "Pré-Termo" OR "Recém-Nascido Pré-Termo" OR "Recém-Nascidos Prematuros" OR "Recém-Nascidos Pré-Termo" OR "Recien Nacido Prematuro" OR "Lactante Nacido Prematuramente" OR "Lactante Nacido Pretérmino" OR "Lactante Prematuro" OR "Lactante Pretérmino" OR "Lactantes Nacidos Prematuramente" OR "Lactantes Nacidos Prematuros" OR "Lactantes Nacidos Pretérmino" OR "Lactantes Prematuros" OR "Lactantes Pretérmino" OR "Neonato Pretérmino" OR "Neonatos Pretérmino" OR prematuridad OR "Prematuridad Neonatal" OR pretérmino OR "Recien Nacido Pretérmino" OR "Recien Nacidos Prematuros" OR "Recien Nacidos Pretérmino" OR "Recém-Nascido de Baixo Peso" OR "Baixo Peso ao Nascer" OR "Recién Nacido de Bajo Peso" OR "recién nacido con peso bajo" OR "Recém-Nascido de muito Baixo Peso" OR "Muito Baixo Peso ao Nascer" OR "Peso muito Baixo ao Nascer" OR "Recién Nacido de muy Bajo Peso" OR "Recién Nacido de muy Bajo Peso" OR "Peso muy Bajo al Nacer" OR "neonato con muy bajo peso" OR "neonato de muy bajo peso" OR "recién nacido con muy bajo peso" OR "Recém-Nascido de Peso Extremamente Baixo ao Nascer" OR "Peso Extremamente Baixo ao Nascer" OR "Recém-Nascido de Extremo Baixo Peso" OR "Recien Nacido con Peso al Nacer Extremadamente Bajo" OR "Peso Extremamente Bajo al Nacer" OR "recién nacido de peso extremadamente bajo" OR "neonato con peso extremadamente bajo" OR "recién nacido de extremado bajo peso" OR "Recém-Nascido Pequeno para a Idade Gestacional" OR "Recién Nacido Pequeño para la Edad Gestacional" OR "recién nacido pequeño para su edad gestacional") AND ("Procedural Pain" OR "Acute Pain" OR "Acute Pains" OR pain OR pains OR ache OR aches OR "Physical Suffering" OR "Physical Sufferings" OR "Dor Processual" OR "Dolor Asociado a Procedimientos Médicos" OR "Dor Aguda" OR "Dolor Agudo" OR dor OR dolor OR "Sofrimento Físico" OR "Sufrimiento Físico") AND ("Pain Management" OR "Pain Managements" OR "Pain Measurement" OR "Pain Measurements" OR "Pain Assessment" OR "Pain Assessments" OR "Pain Intensity" OR "Pain Intensities" OR "Pain Severity" OR "Pain Severities" OR "Analgesia Tests" OR "Analgesia Test" OR "Pain Scale" OR "Pain Scales" OR "Pain Test" OR "Pain Tests" OR "Nociception Tests" OR "Nociception Test" OR "Formalin Test" OR "Formalin Tests" OR "Manejo da Dor" OR "Manejo del Dolor" OR "tratamiento del dolor" OR "Medição da Dor" OR "Avaliação da Dor" OR "Escala de Dor" OR "Escala da Dor" OR "Gravidade da Dor" OR "Intensidade da Dor" OR "Questionário da Dor" OR "Teste da Dor" OR "Teste da Formalina" OR "Teste do Torniquete" OR "Testes de Analgesia" OR "Testes de Nocicepção" OR "Dimensión del Dolor" OR "medida del dolor" OR "Cuestionario de Dolor" OR "Escala del Dolor" OR "Escala de Dolor" OR "Evaluación del Dolor" OR "Intensidad del Dolor" OR "Severidad del Dolor" OR "Test de Dolor" OR "Test de Formalina" OR "Tests de Analgesia" OR "Tests de Nocicepción" OR "prueba de formalina" OR "pueba de dolor") AND (therapeutics OR treatment OR treatments OR therapies OR therapy OR "Kangaroo-Mother Care Method" OR "Kangaroo Mother Care Method" OR "Kangaroo Mother Care Methods" OR "Kangaroo Mother Care" OR "Kangaroo-Mother Care" OR "kangaroo mother method" OR "kangaroo care" OR "skin to skin care" OR "skin-to-skin" OR "skin to skin" OR "skin to skin contact" OR terapêutica OR terapêuticas OR "Procedimento Curativo" OR terapia OR tratamento OR "Procedimentos Curativos" OR terapêuticos OR terapias OR tratamentos OR "Método Canguru" OR "Mãe Canguru" OR "Método Mãe Canguru" OR "Método Mãe-Canguru" OR "Método Madre-Canguro" OR "Madre Canguro" OR "método de la madre canguro") AND ( db:("LILACS")) | 239 |
| ProQuest Dissertations & Theses Global (PQDT Global) | noft("Newborn Infant" OR "Newborn Infants" OR Newborns OR Newborn OR Neonate OR Neonates OR baby OR babies OR "Premature Infant" OR "Preterm Infants" OR "Preterm Infant" OR "Premature Infants" OR "Neonatal Prematurity" OR premature OR preterm OR prematurity OR "Low Birth Weight Infant" OR "Low-Birth-Weight Infant" OR "Low-Birth-Weight Infants" OR "Low Birth Weight" OR "Low Birth Weights" OR "Very Low Birth Weight Infant" OR "Very-Low-Birth-Weight Infant" OR "Very-Low-Birth-Weight Infants" OR "Very Low Birth Weight" OR "Extremely Low Birth Weight Infant" OR "Infant Small for Gestational Age" OR "Small for Gestational Age Infant") AND noft("Procedural Pain" OR "Acute Pain" OR "Acute Pains" OR Pain OR Pains OR Ache OR Aches OR "Physical Suffering" OR "Physical Sufferings") AND noft("Pain Management" OR "Pain Managements" OR "Pain Measurement" OR "Pain Measurements" OR "Pain Assessment" OR "Pain Assessments" OR "Pain Intensity" OR "Pain Intensities" OR "Pain Severity" OR "Pain Severities" OR "Analgesia Tests" OR "Analgesia Test" OR "Pain Scale" OR "Pain Scales" OR "Pain Test" OR "Pain Tests" OR "Nociception Tests" OR "Nociception Test" OR "Formalin Test" OR "Formalin Tests") AND noft(therapeutics OR treatment OR treatments OR therapies OR therapy OR "Kangaroo-Mother Care Method" OR "Kangaroo Mother Care Method" OR "Kangaroo Mother Care Methods" OR "Kangaroo Mother Care" OR "Kangaroo-Mother Care" OR "kangaroo mother method" OR "kangaroo care" OR "skin to skin care" OR "skin-to-skin" OR "skin to skin" OR "skin to skin contact") | 55 |
| Google Scholar | (Newborns OR Newborn OR Neonate OR Neonates OR premature OR preterm OR "Recém-Nascido" OR "Recém-Nascidos") AND ("Procedural Pain" OR "Acute Pain" OR Pain OR Dor OR Dolor) AND ("Pain Management" OR "Pain Measurement" OR "Pain Assessment" OR "Manejo da Dor" OR "Manejo del Dolor") AND ("Kangaroo-Mother Care Method" OR "Kangaroo Mother Care Method" OR "kangaroo mother method" OR "kangaroo care" OR "skin to skin" OR Terapia OR Tratamento OR "Método Canguru" OR "Mãe Canguru OR therapeutics OR treatment OR therapy) | 100 |

Search strategies were performed for each database by using specifics words combinations and truncations with the support of a librarian.
