# Supplementary material for: Impact of skin-to-skin contact on acute procedural pain in newborns: a systematic review and meta-analysis
Source: J Pediatr (Rio J). 2025 Sep 11;101(6):101442. doi: 10.1016/j.jped.2025.101442 (PMC12744622; doi:10.1016/j.jped.2025.101442)
Supplement: Supplementary file 2 [file mmc2.docx]

**JPED-D-25-00185_Supplementary Material**

**Supplementary Table 1**. Description of the studies included in the review.

| **Author, year** | **Country** | **Register** | **Study design** | **Intervention, Control, Sample size** | **Gestational age at birth** | **Maximum follow-up time** | **Main conclusion** |
| --- | --- | --- | --- | --- | --- | --- | --- |
| Gabriel et al., 2013^21^ | Spain | NCT01576432 | Randomized controlled trial | Total: 136  STS: 31  SC: 32 | Between 37 and 41 weeks | Up to 2 minutes | The administration of a carbohydrate solution was more effective than skin-to-skin contact alone in reducing pain during heel puncture in full-term newborns, as demonstrated by lower scores on the NIPS scale and less crying time in the group that received the sugary solution. However, both methods had limitations when used in isolation, being less effective than the combined strategies. |
| Okan et al., 2010^31^ | Türkiye | - | Randomized controlled clinical trial | Total: 107  STS: 36  C: 36 | Mean of 39.5 weeks | Up to 5 minutes | Skin-to-skin contact with the mother, performed before, during, and after the heel puncture, significantly reduced physiological signs of pain (increased heart rate and decreased oxygen saturation) and behavioral signs (crying time and pain facial expressions) compared to the control group without contact. Pain was assessed using the NFCS, which showed lower scores in the skin-to-skin contact group. |
| Chang et al., 2020^15^ | United States | NCT03421158 | Randomized controlled clinical trial | Total: 226  STS: 38  C: 50  CHO: 42  NNS: 51  B: 45 | Between 38 and 40 weeks | Until the completion of the procedure | All non-pharmacological interventions were significantly more effective in reducing neonatal pain compared to the control group (no intervention), showing lower scores on the NPASS scale. Carbohydrate solution demonstrated the greatest analgesic effect according to the NPASS score, outperforming skin-to-skin contact but showing no statistical difference compared to breastfeeding or non-nutritive sucking. Skin-to-skin contact was the least effective among the interventions, although still better than the control. All methods also significantly reduced crying time and procedure duration. |
| Fallah et al., 2016^20^ | Iran | RCT201505142639N16 | Parallel, single-arm, unblinded randomized controlled trial. | Total: 120  STS: 40  S: 40  B: 40 | Between 37 and 42 weeks | Up to 2 minutes | Breastfeeding was the most effective intervention in reducing pain associated with BCG vaccination, showing lower scores on the NIPS scale during and after the procedure, a higher frequency of vaccinations with minimal pain, and a shorter crying time. Skin-to-skin contact and swaddling also significantly reduced pain compared to no intervention but were less effective than breastfeeding. |
| Gholami et al., 2021^22^ | Iran | IRCT20180108038265N2 | Double-blind, randomized, controlled clinical trial | Total: 90  STS: 30  C: 30 | Between 28 and 36 weeks. | From the time of newborn stabilization until discharge from the Neonatal Intensive Care Unit (NICU). | Skin-to-skin contact performed by mothers of preterm infants in the neonatal intensive care unit (NICU) was significantly more effective than standard care (control group) in reducing neonatal pain, as assessed by the NIPS. |
| Soltani et al., 2008^37^ | Iran | IRCT20151201253256N1 | Double-blind, randomized, controlled clinical trial | Total: 161  STS: 38  CHO: 41  B: 42 | Between 37 and 42 weeks | Until the completion of the procedure | Among the three non-pharmacological interventions evaluated, breastfeeding was the most effective strategy in reducing neonatal pain, with the lowest score on the NIPS scale, followed by carbohydrate solution, and finally, skin-to-skin contact. |
| Chidambaram et al., 2013^17^ | India | - | Randomized, crossover clinical trial | Total: 100  STS: 50  C: 50 | Between 32 and 36 weeks | 30 minutes | Skin-to-skin contact was significantly more effective than standard care (control) in reducing pain in preterm infants, as measured by the PIPP scale. Pain scores were lower at 15 and 30 minutes after heel lance in the skin-to-skin group compared to the control group. |
| Liao et al., 2020^27^ | China | - | Randomized controlled clinical trial | Total: 78  STS: 39  C: 39 | Between 37 and 42 weeks | 42 days after | Skin-to-skin contact was significantly more effective than standard care in reducing neonatal pain, as assessed by the NIPS during painful procedures such as heel lance. |
| Sen et al., 2020^35^ | Türkiye | - | Assessor-blinded randomized controlled trial | Total: 74  STS: 23  CHO: 25 | Between 32 and 37 weeks | 2 minutes | Both skin-to-skin contact and carbohydrate solution were effective in reducing pain during heel lance in preterm infants, as assessed by the PIPP scale. However, the skin-to-skin contact group showed significantly lower pain scores after the procedure, demonstrating greater efficacy compared to the carbohydrate solution. |
| Yýldýzdaþ et al., 2022^40^ | Türkiye | - |  | Total: 64  STS: 32  CHO: 32 | Between 34 and 41 weeks | Up to 15 seconds | Skin-to-skin contact and the administration of carbohydrate solution showed similar effects in reducing pain during venipuncture in newborns, according to the NIPS. Although the group that received the carbohydrate solution had slightly lower pain scores and shorter crying time, the differences were not statistically significant. |
| Wang et al., 2020^38^ | China | - | Randomized controlled clinical trial | Total: 76  STS: 36  C: 37 | From 31 to 33 weeks | 48 hours | Skin-to-skin contact was significantly more effective than standard care in reducing pain — measured by the PIPP — during repeated heel pricks in preterm infants. The skin-to-skin group showed lower pain scores, lower heart rate, higher oxygen saturation, and better brain oxygenation stability (rcSO₂ and cFTOE) compared to the control group. |
| Patel et al., 2022^34^ | India | CTRI/2020/01/022984 | Randomized controlled clinical trial | Total: 76  STS: 36  S: 37 | 39 weeks | 2 minutes | Skin-to-skin contact was significantly more effective than swaddling in reducing neonatal pain after the vitamin K injection, as assessed by the NIPS. Although both groups showed an increase in pain immediately after the injection, the newborns in the skin-to-skin contact group showed a significant reduction in pain at 2 minutes post-procedure, while in the swaddling group, pain remained elevated. |
| Pasquier et al., 2013^33^ | Canada | - | Assessor-blinded randomized controlled trial | Total: 60  STS: 30  C: 30 | > 37 weeks | Up to 60 minutes | Immediate skin-to-skin contact after cesarean delivery significantly reduced neonatal pain and stress associated with the vitamin K injection, compared to the control group that received standard care. |
| Pandita et al., 2018^32^ | India | - | Assessor-blinded randomized controlled trial | Total: 61  STS: 32  S: 29 | Between 34 and 42 weeks | Up to 5 minutes | Skin-to-skin contact was significantly more effective than swaddling in reducing pain associated with intramuscular vaccination in infants. Babies in the skin-to-skin contact group had lower NIPS pain scores at 1 and 5 minutes after the injection and shorter crying time compared to the swaddling group. |
| Xiaomei Cong, 2006^39^ | United States | - | Randomized crossover clinical trial | Total: 28  STS: 26  C: 25 | Between 30 and 32 weeks | 80 minutes in study 1, 30 minutes in study 2 | Skin-to-skin contact was significantly more effective than standard care (incubator) in reducing behavioral and physiological pain during and after heel prick in preterm newborns. Evaluated by the Premature Infant Pain Profile (PIPP), pain was on average 27% lower during the procedure and up to 25% lower in the recovery phase in the skin-to-skin contact group. |
| Nimbalkar et al., 2012^30^ | India | - | Randomized controlled, double-blind, crossover trial | Total: 50  STS: 19  C: 28 | Between 32 and 36 weeks | Up to 15 minutes | Skin-to-skin contact for just 15 minutes before heel lance was significantly more effective than standard care in reducing pain in preterm newborns, as assessed by the PIPP. |
| Johnston et al., 2003^23^ | Canada | - | Assessor-blinded randomized controlled crossover trial | Total: 50  STS: 19  C: 28 | Between 32 and 36 weeks | Up to 2 minutes | Maternal skin-to-skin contact was significantly more effective than standard incubator care in reducing pain during heel prick in preterm newborns. Pain scores, assessed using the PIPP, were 2 points lower in the skin-to-skin group in the first 90 seconds after the procedure, representing a clinically relevant difference. |
| Celeste et al., 2008^14^ | Canada | SRCTN63551708 | Randomized crossover clinical trial | Total: 75  STS: 31  C: 30 | Between 28 and 31 weeks | Up to 2 minutes | Skin-to-skin contact was significantly more effective than standard incubator care in reducing pain and promoting physiological recovery after heel prick in very preterm newborns. Pain scores (PIPP) were significantly lower in the skin-to-skin group starting 90 seconds after the procedure, with a reduction of about 2 points. Additionally, heart rate recovery time was 70 seconds faster in the skin-to-skin group. |
| Mendoza et al., 2012^28^ | Spain | - | Assessor-blinded randomized controlled crossover trial | Total: 136  STS: 33  CHO: 33 | - | Up to 5 minutes | Both skin-to-skin contact and oral administration of carbohydrate solution were effective in reducing pain during painful procedures in neonates, as assessed by the NIPS scale. However, the carbohydrate solution demonstrated greater efficacy, showing lower pain scores, lower heart rate, and shorter crying time compared to skin-to-skin contact. |
| Kapoor et al., 2021^24^ | India | CTRI/2017/11/010612 | Non-blind randomized controlled trial with parallel groups | Total: 160  STS: 45  C: 50  CHO: 54 | Mean 36 weeks | Up to 5 minutes | Skin-to-skin contact and carbohydrate solution were equally effective in reducing pain in late preterm newborns undergoing heel prick, with similar scores on the PIPP scale. Both groups also had significantly less crying time compared to the control group. |
| Shukla et al., 2017^36^ | India | - | Randomized controlled clinical trial | Total: 100  STS: 50  CHO: 50 | Between 26 and 36 weeks | - | The carbohydrate solution was significantly more effective than skin-to-skin contact in reducing pain in premature neonates during heel puncture. The neonates who received the carbohydrate solution had lower pain scores on the PIPP scale, lower heart rate, and shorter crying duration compared to the skin-to-skin group. |
| Chermont et al., 2009^16^ | Brazil | NCT00713986 | Randomized controlled trial with partial blinding | Total: 640  STS: 160  C: 160  CHO: 160 | Mean of 39 weeks | Up to 2 minutes | Both skin-to-skin contact and carbohydrate solution were effective in reducing pain in term neonates during intramuscular hepatitis B vaccination when compared to the control group (no analgesia). However, only skin-to-skin contact alone significantly reduced pain scores (PIPP) compared to the control group, while carbohydrate solution alone had no significant effect on PIPP scores. |
| Karakus Türker et al., 2022^25^ | Türkiye | - | Randomized controlled clinical trial | Total: 70  STS: 35  B: 35 | ≥ 37 weeks | Immediately after the procedure | Both skin-to-skin contact and breastfeeding were effective in reducing pain in newborns undergoing vaccination. However, there was no statistically significant difference between the groups regarding pain scores (NIPS), heart rate, oxygen saturation, and crying time after the injection. |
| Akcan et al., 2009^12^ | Türkiye | - | Randomized controlled clinical trial | Total: 50  STS: 25  C: 25 | Between 26 and 36 weeks | Up to 10 minutes | Skin-to-skin contact initiated 30 minutes before and maintained for 10 minutes after the puncture was significantly more effective than standard care (control group) in reducing pain in preterm newborns during invasive procedures, as assessed by the PIPP scale. |
| Mosayebi et al., 2014^29^ | Iran | - | Randomized controlled clinical trial | Total: 64  STS: 32  C: 32 | Between 30 and 36 weeks | Up to 2 minutes | Skin-to-skin contact, initiated 15 minutes before and maintained during and after the heel prick, was significantly more effective than standard care (control group) in the incubator in reducing pain in preterm newborns, as assessed by the PIPP scale. |
| Kaya et al., 2023^26^ | Türkiye | IRCT20200115046139N1 | Randomized controlled clinical trial | Total: 60  STS: 30  C: 30 | Between 37 and 41 weeks | Up to 10 minutes | Maternal skin-to-skin contact, initiated 5 minutes before and maintained for 5 minutes after the intramuscular vitamin K injection, was significantly more effective than standard care (control group) in reducing pain in newborns, as assessed by the NIPS scale. |
| Çiftci et al., 2022^18^ | Türkiye | - | Randomized controlled clinical trial | Total: 148  STS: 37  C: 37  S: 37 | Between 32 and 37 weeks | - | Skin-to-skin contact was significantly more effective than standard care (no intervention) in reducing pain and increasing comfort in preterm newborns during peripheral vascular access, as measured by the NIPS and PICS scales. Swaddling showed intermediate results. |
| Dezhdar et al., 2016^19^ | Iran | IRCT2014042212830N3R1 | Randomized controlled clinical trial | Total: 90  STS: 25  C: 29  S: 38 | Less than 37 weeks | - | Both skin-to-skin contact and swaddling were significantly more effective than the control group in reducing pain during venous puncture in preterm newborns, as assessed by the PIPP scale. |
| Castral et al., 2008^13^ | Brazil, Canada | - | Randomized controlled clinical trial | Total: 59  STS: 31  C: 28 | Mean of 35 weeks (skin-to-skin contact group) and 36 weeks (control group) | Immediately after the procedure | Skin-to-skin contact was significantly more effective than standard care (control group) in reducing pain in premature newborns undergoing venous puncture, as measured by pain scores on the PIPP scale. |

Legend: B: Breastfeeding; C: Control (no intervention); cFTOE: Cerebral tissue oxygen extraction fraction; CHO: Carbohydrate solution (e.g., sucrose, dextrose); EBM: Expressed Breast Milk; NFCS: Neonatal Facial Coding System; NICU: Neonatal Intensive Care Unit; NIPS: Neonatal Infant Pain Scale; NNS: Non-nutritive sucking; NPASS: Neonatal Pain, Agitation, and Sedation Scale; P: Pacifier (non-nutritive sucking); PIPP: Premature Infant Pain Profile; rcSO₂: Regional cerebral oxygen saturation; S: Swaddling; STS: Skin-to-skin; VLBW: Very Low Birth Weight.
