# Supplementary material for: Impact of skin-to-skin contact on acute procedural pain in newborns: a systematic review and meta-analysis
Source: J Pediatr (Rio J). 2025 Sep 11;101(6):101442. doi: 10.1016/j.jped.2025.101442 (PMC12744622; doi:10.1016/j.jped.2025.101442)
Supplement: Supplementary file 3 [file mmc3.docx]

**JPED-D-25-00185_Supplementary Material_Figures**


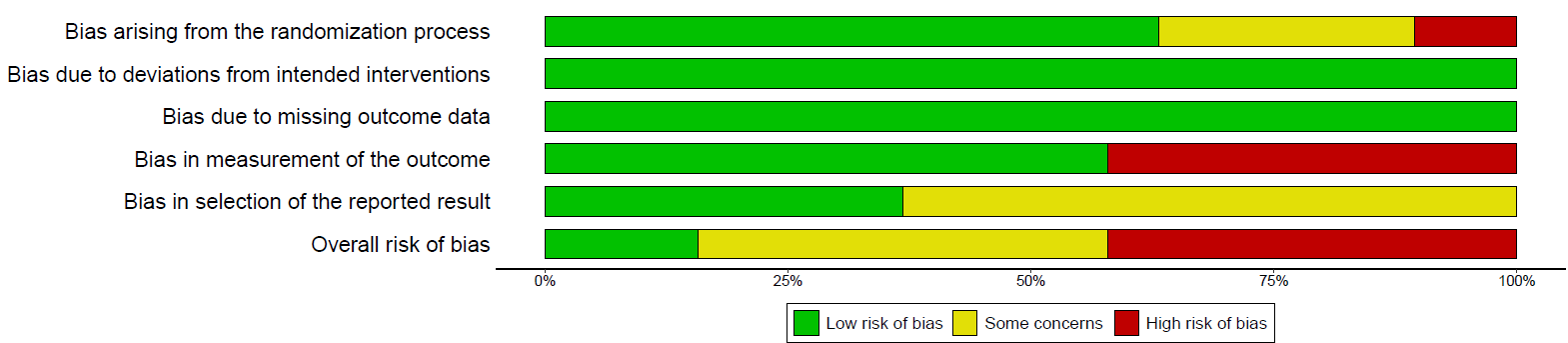


**Supplementary Figure 1** Risk of Bias 2 (RoB 2) Assessment: Skin-to-Skin Contact Versus Control for Procedural Pain in Newborns.


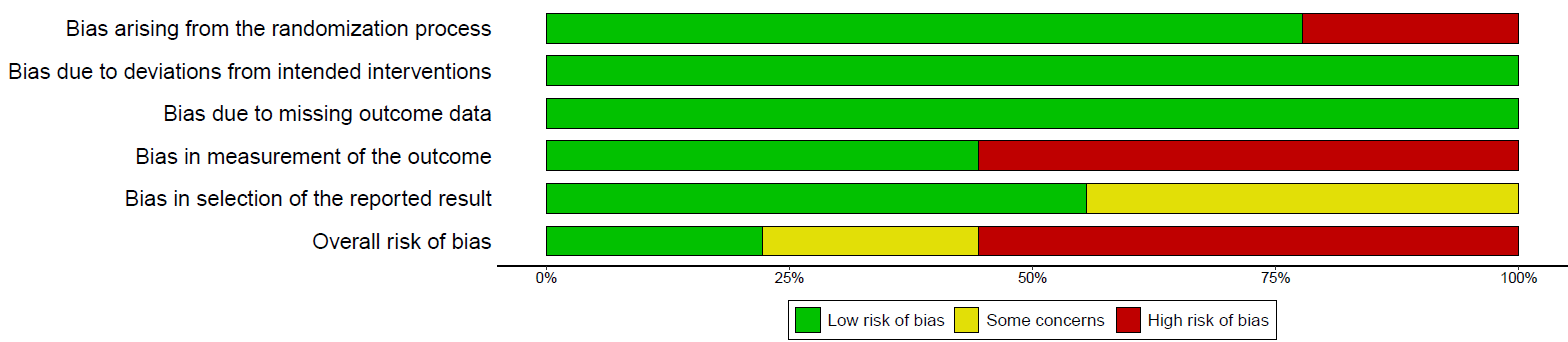


**Supplementary Figure 2** Risk of Bias 2 (RoB 2) Assessment: Skin-to-Skin Contact Versus Carbohydrate Solution for Procedural Pain in Newborns.


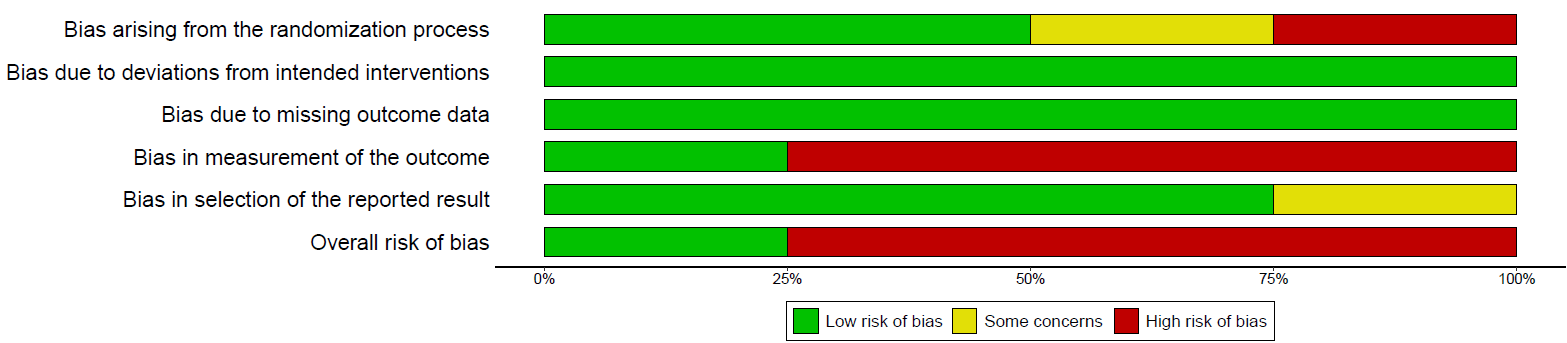


**Supplementary Figure 3** Risk of Bias 2 (RoB 2) Assessment: Skin-to-Skin Contact Versus Breastfeeding for Procedural Pain in Newborns.


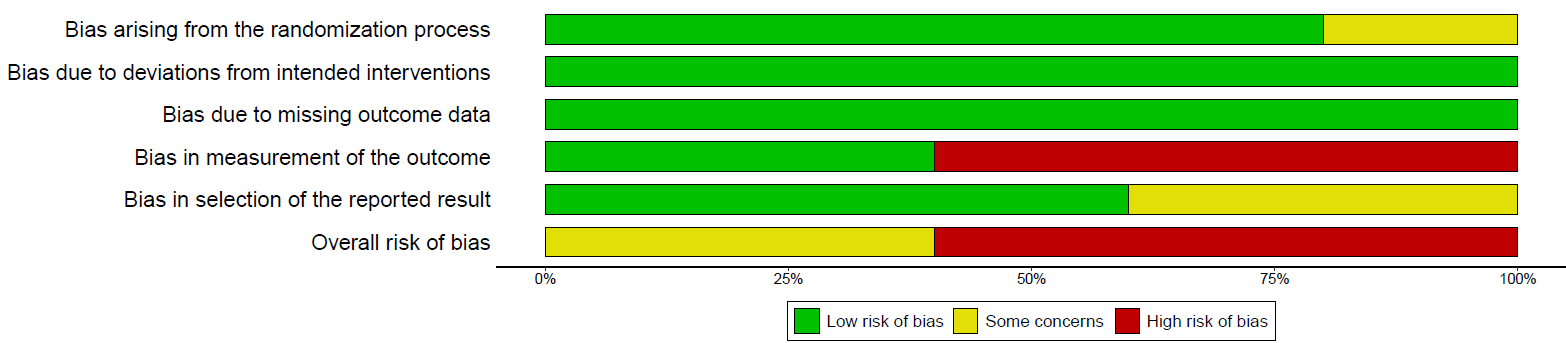


**Supplementary Figure 4** Risk of Bias 2 (RoB 2) Assessment: Skin-to-Skin Contact Versus Swaddling for Procedural Pain in Newborns.


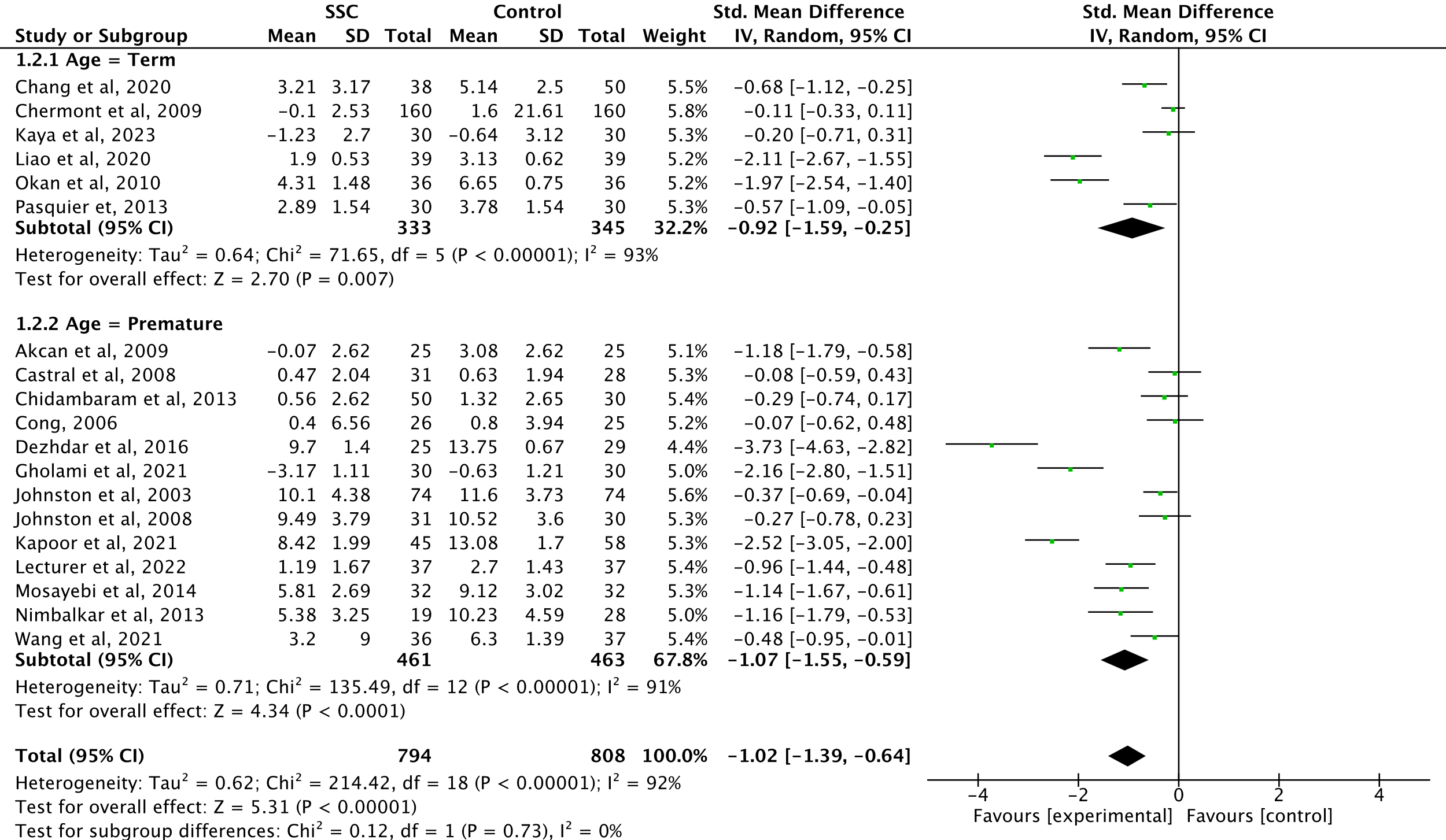


**Supplementary Figure 5** Meta-Analysis: Skin-to-Skin Contact vs. Control for Procedural Pain in Newborns – Subgroup Analysis by Gestational Age.


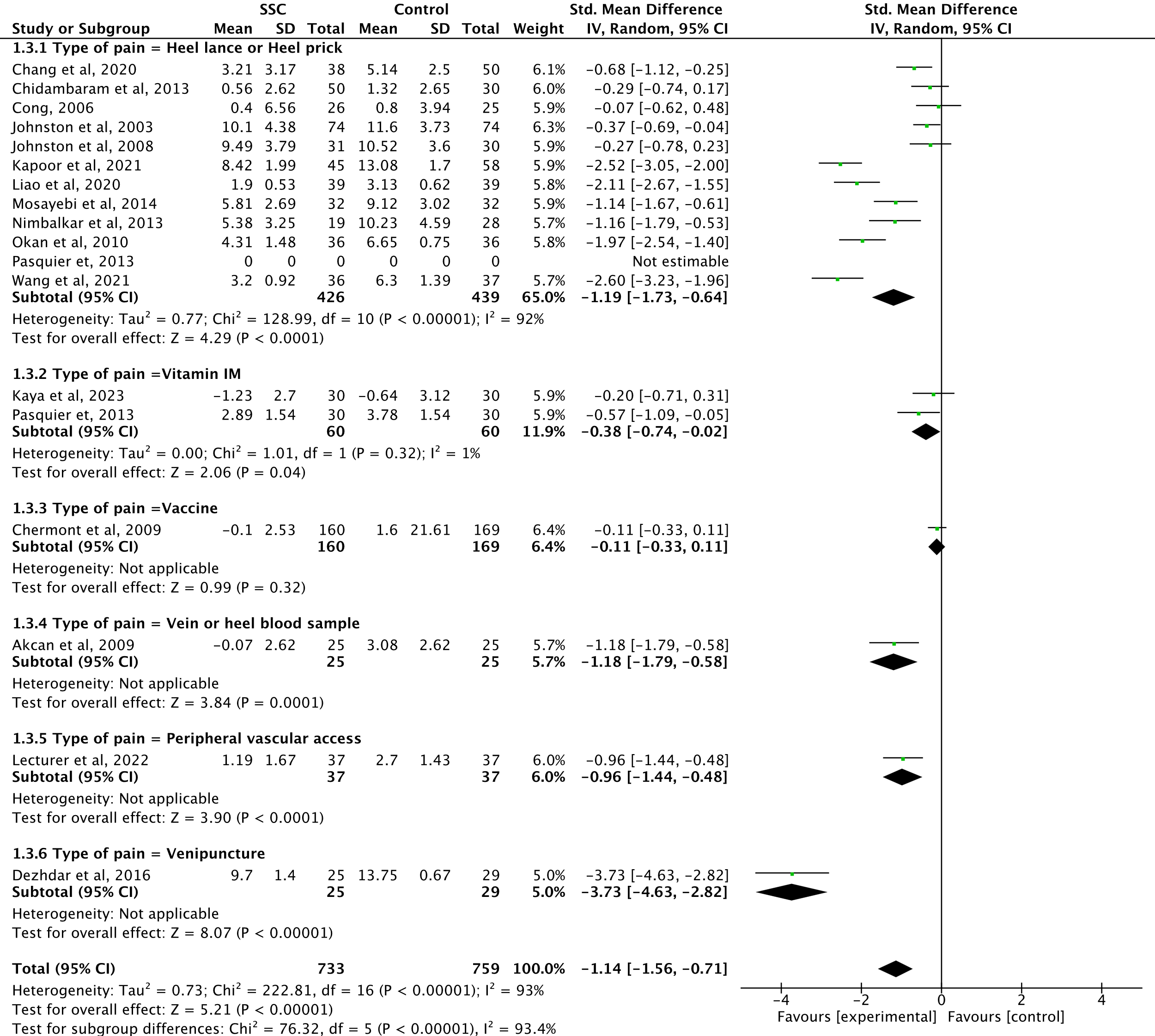


**Supplementary Figure 6** Meta-Analysis: Skin-to-Skin Contact vs. Control for Procedural Pain in Newborns – Subgroup Analysis by Type of Pain.


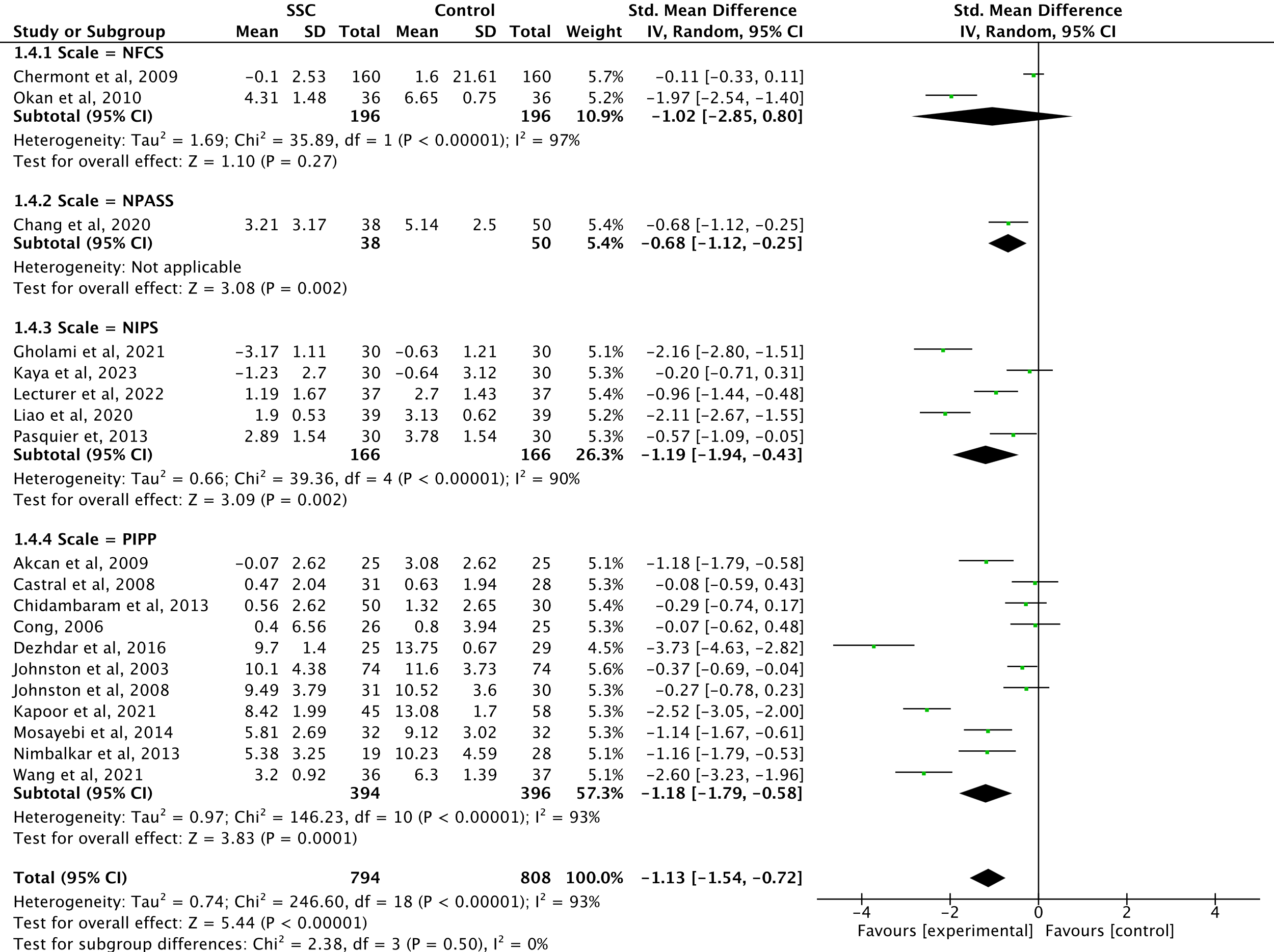


**Supplementary Figure 7** Meta-Analysis: Skin-to-Skin Contact vs. Control for Procedural Pain in Newborns – Subgroup Analysis by Type of Scale.


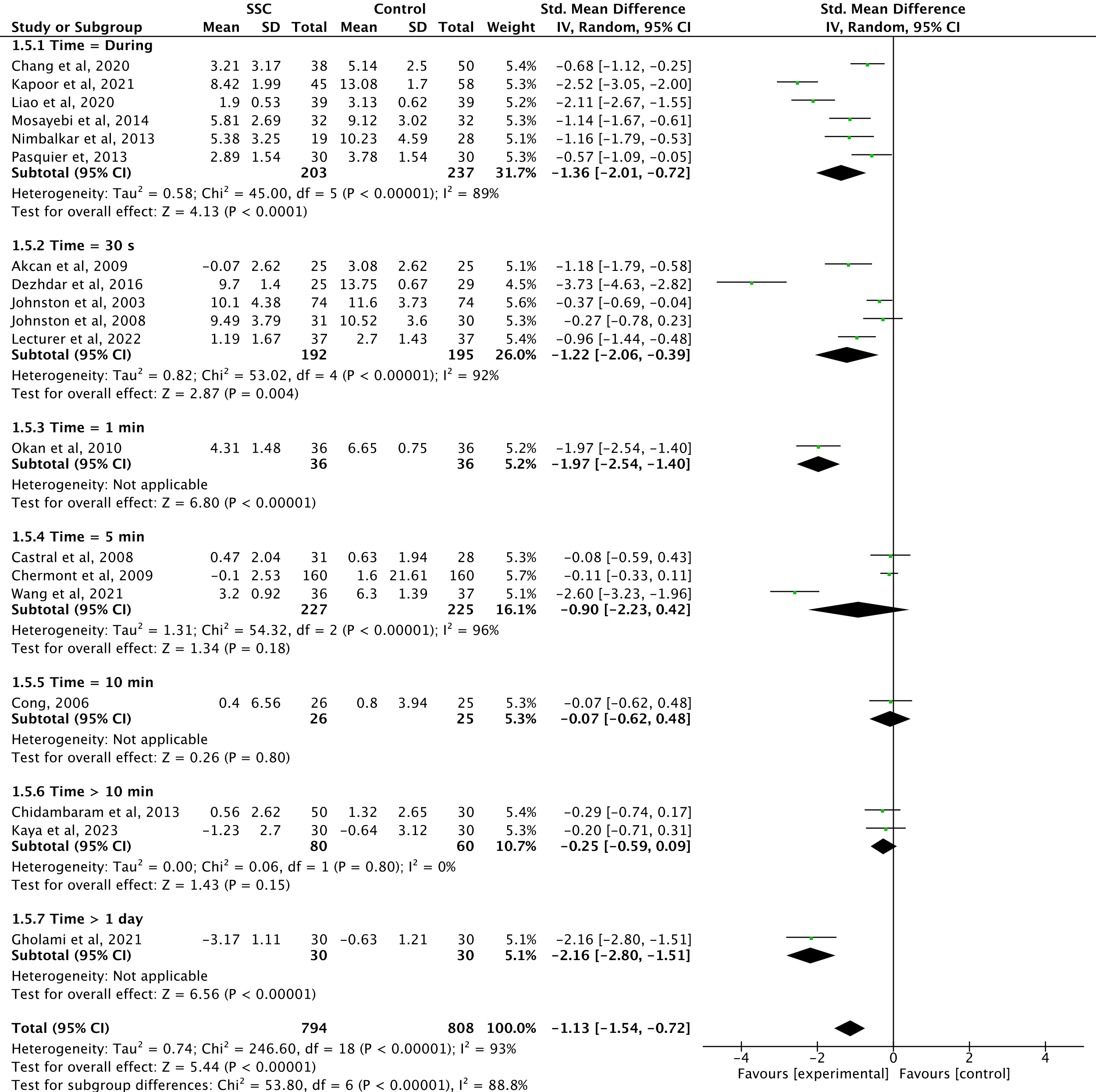


**Supplementary Figure 8** Meta-Analysis: Skin-to-Skin Contact vs. Control for Procedural Pain in Newborns – Subgroup Analysis by Time of Measurement After the Procedure.


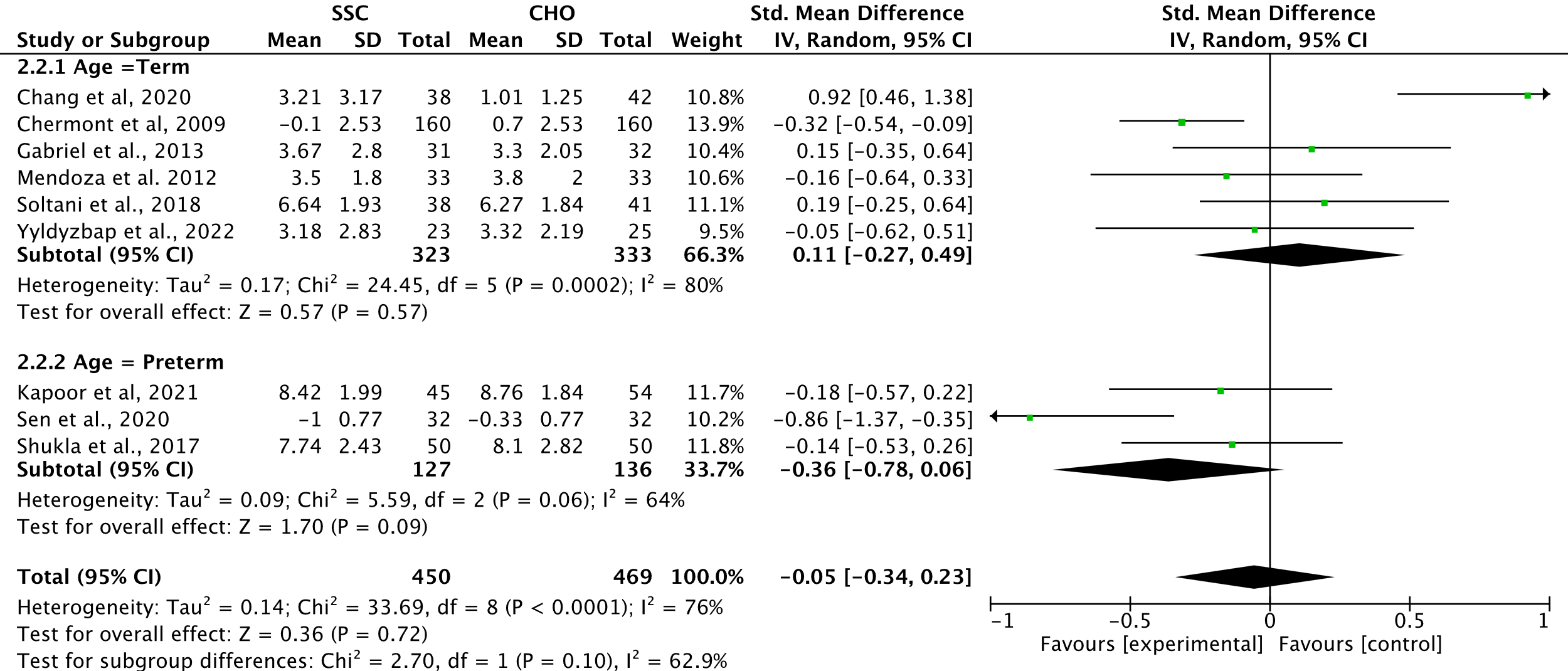


**Supplementary Figure 9** Meta-Analysis: Skin-to-Skin Contact vs Carbohydrate Solution for Procedural Pain in Newborns – Gestational Age Subgroup.


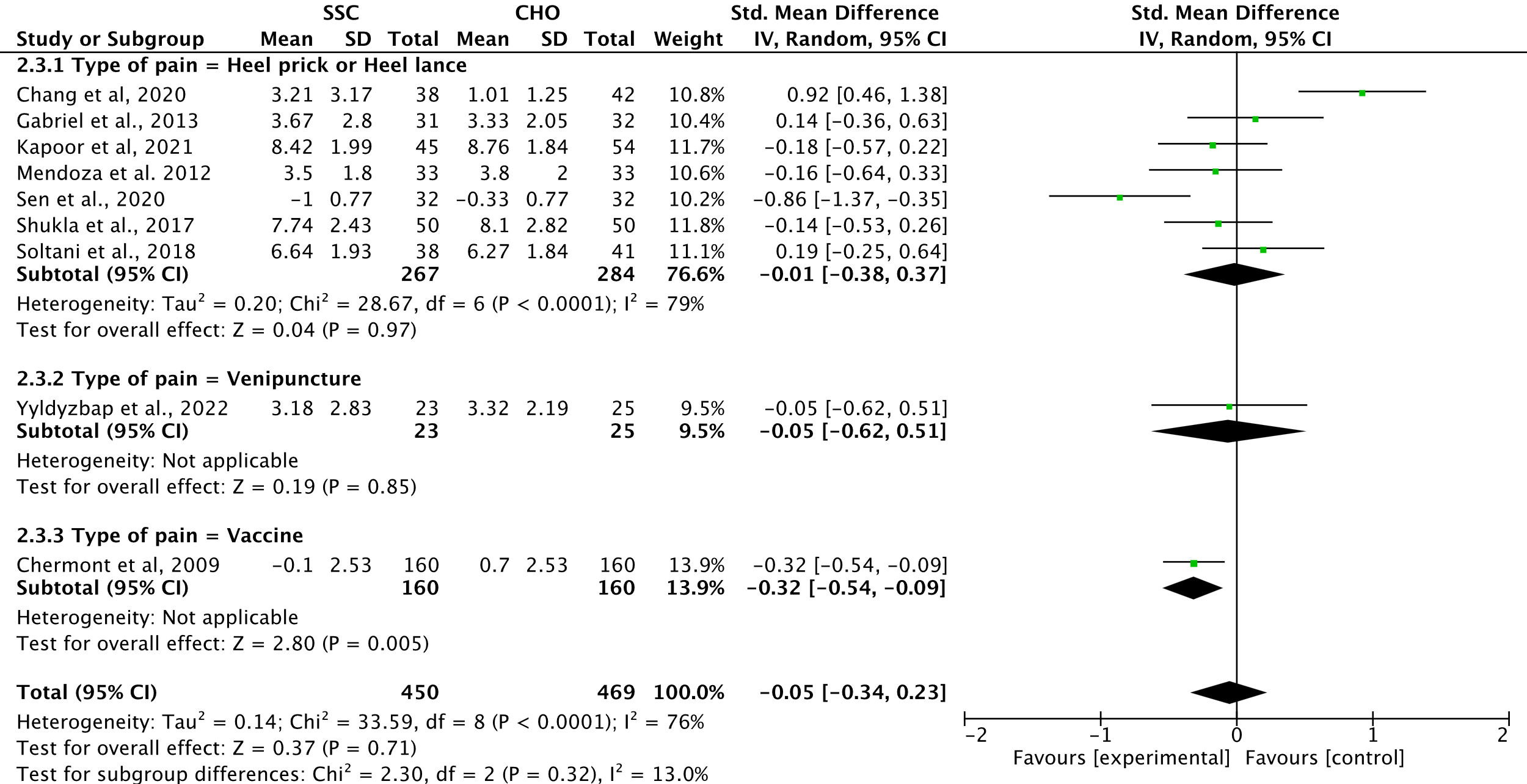


**Supplementary Figure 10** Meta-Analysis: Skin-to-Skin Contact vs Carbohydrate Solution for Procedural Pain in Newborns – Subgroup by Type of Pain.


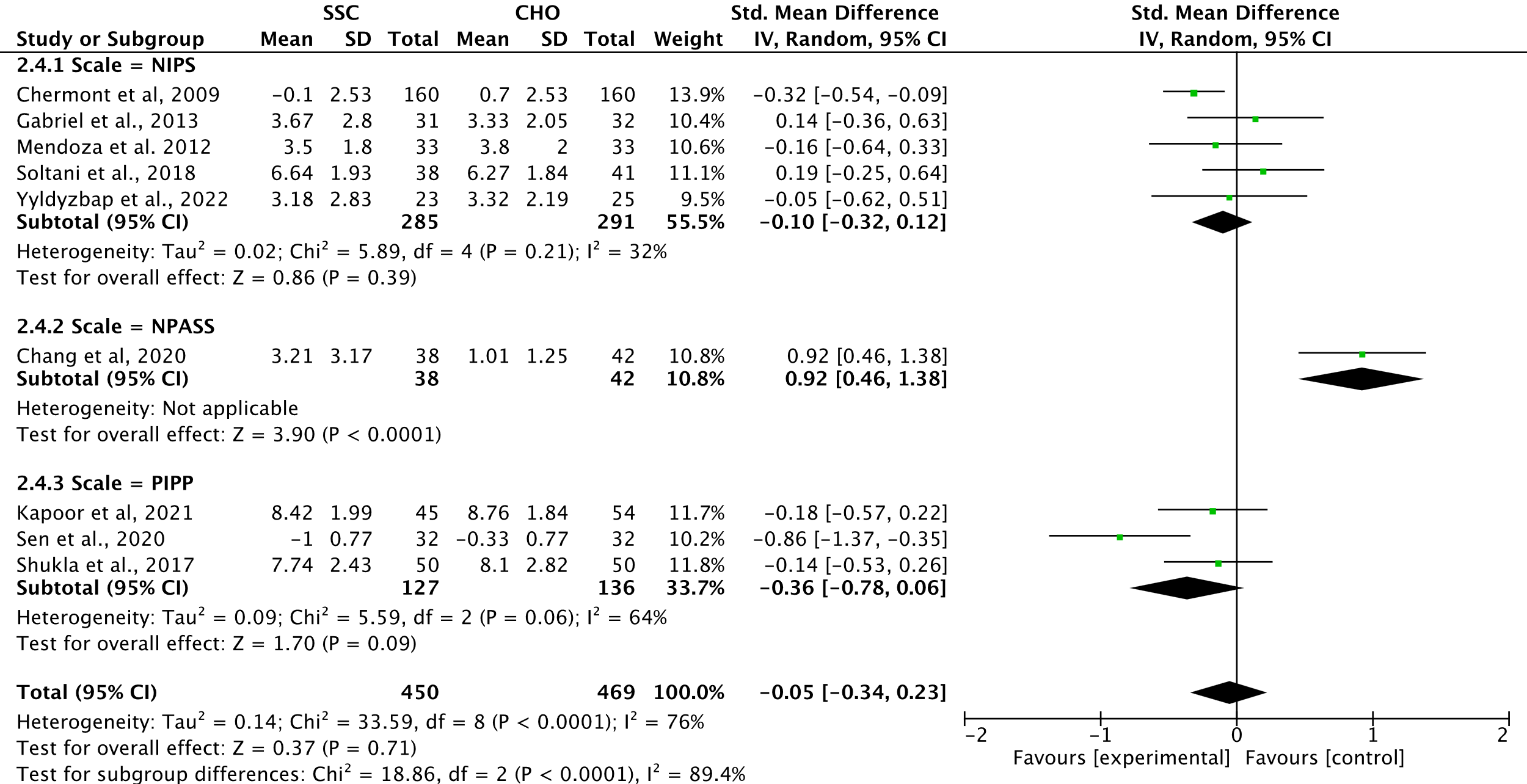


**Supplementary Figure 11** Meta-Analysis: Skin-to-Skin Contact vs Carbohydrate Solution for Procedural Pain in Newborns – Subgroup Analysis by Type of Scale.


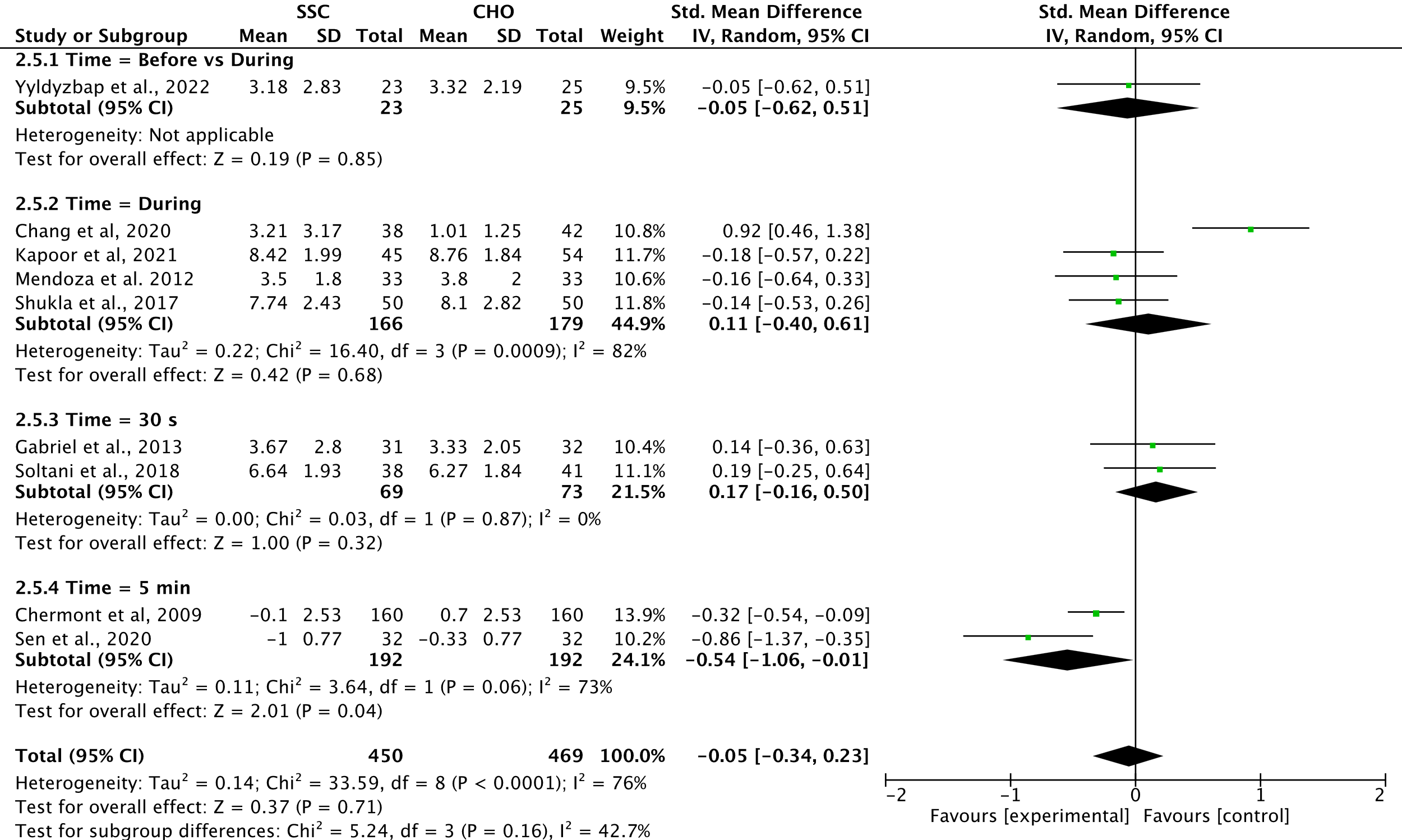


**Supplementary Figure 12** Meta-Analysis: Skin-to-Skin Contact vs Carbohydrate Solution for Procedural Pain in Newborns – Subgroup Analysis by Time of Measurement After the Procedure.


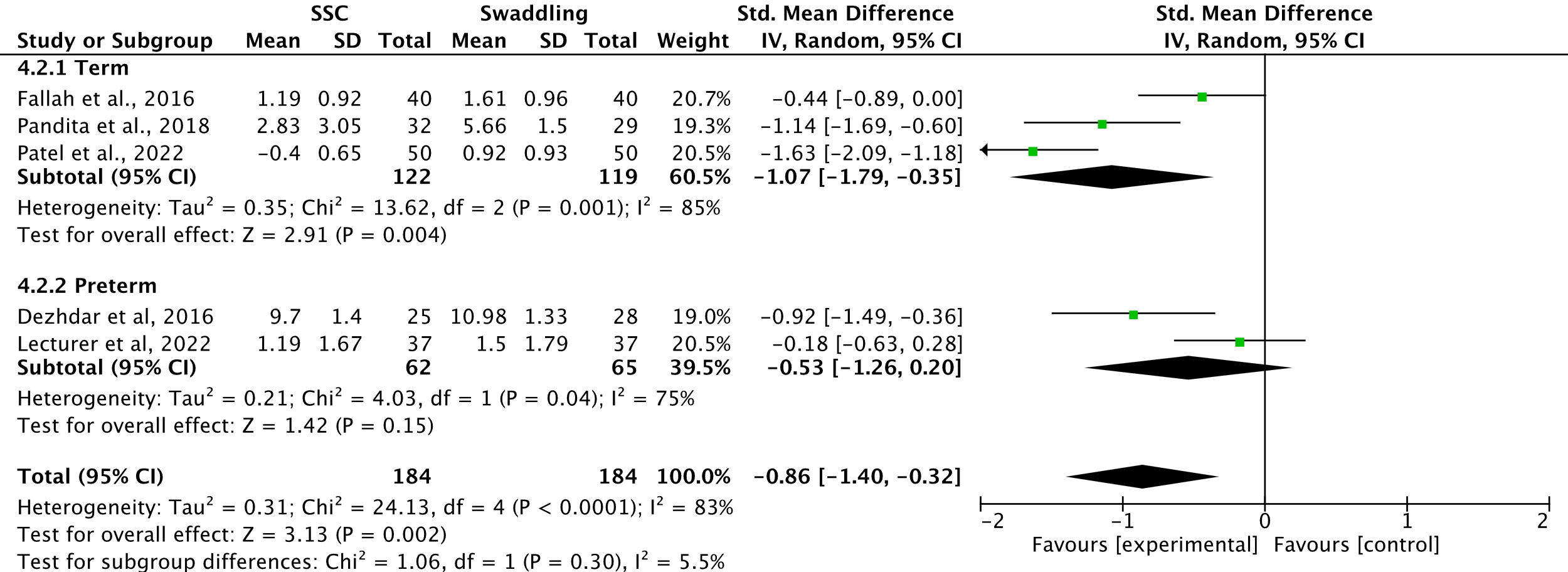


**Supplementary Figure 13** Meta-Analysis: Skin-to-Skin Contact vs. Swaddling for Procedural Pain in Newborns – Gestational Age Subgroup.


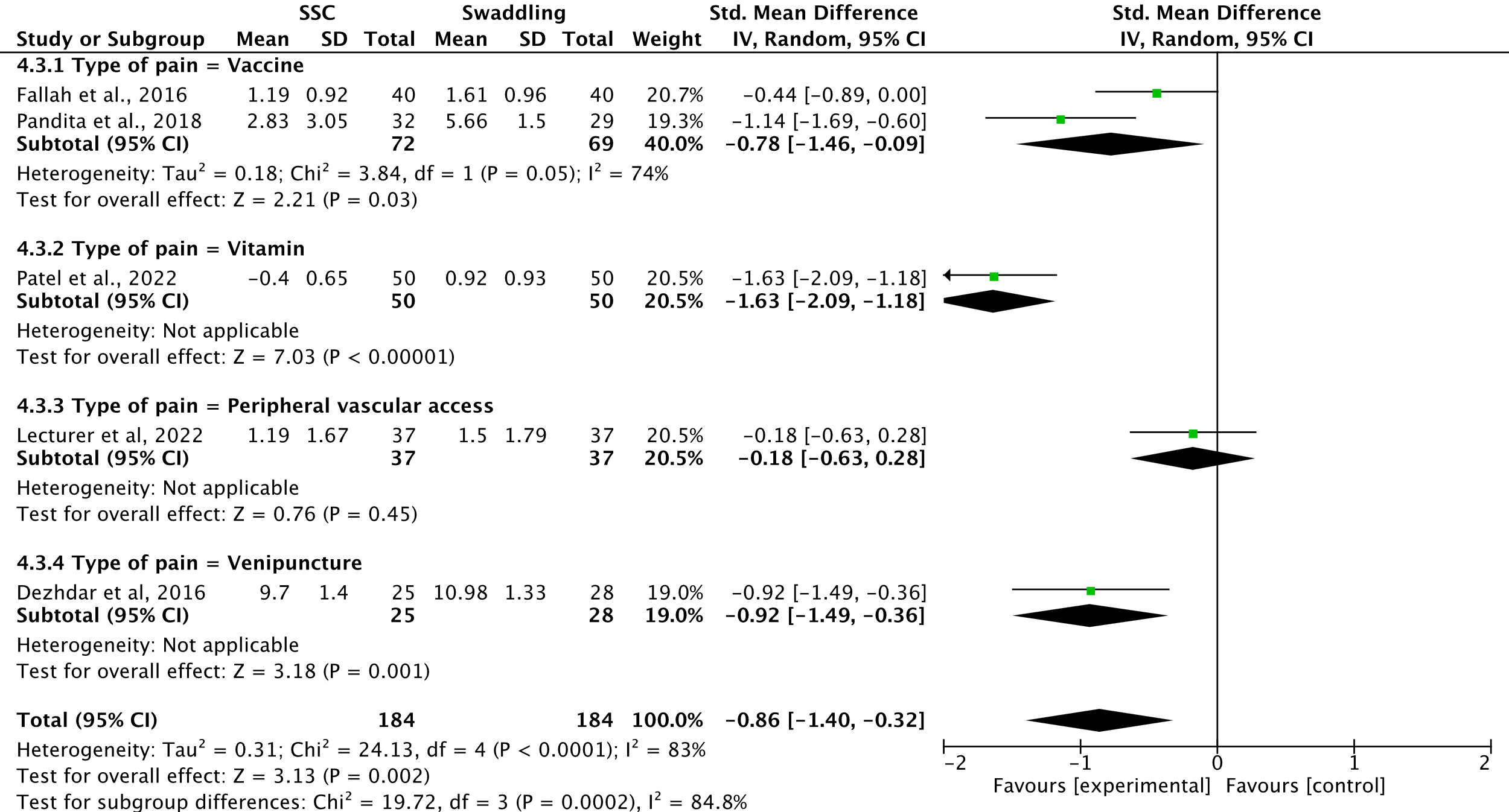


**Supplementary Figure 14** Meta-Analysis: Skin-to-Skin Contact vs. Swaddling for Procedural Pain in Newborns – Subgroup Analysis by Type of Pain.


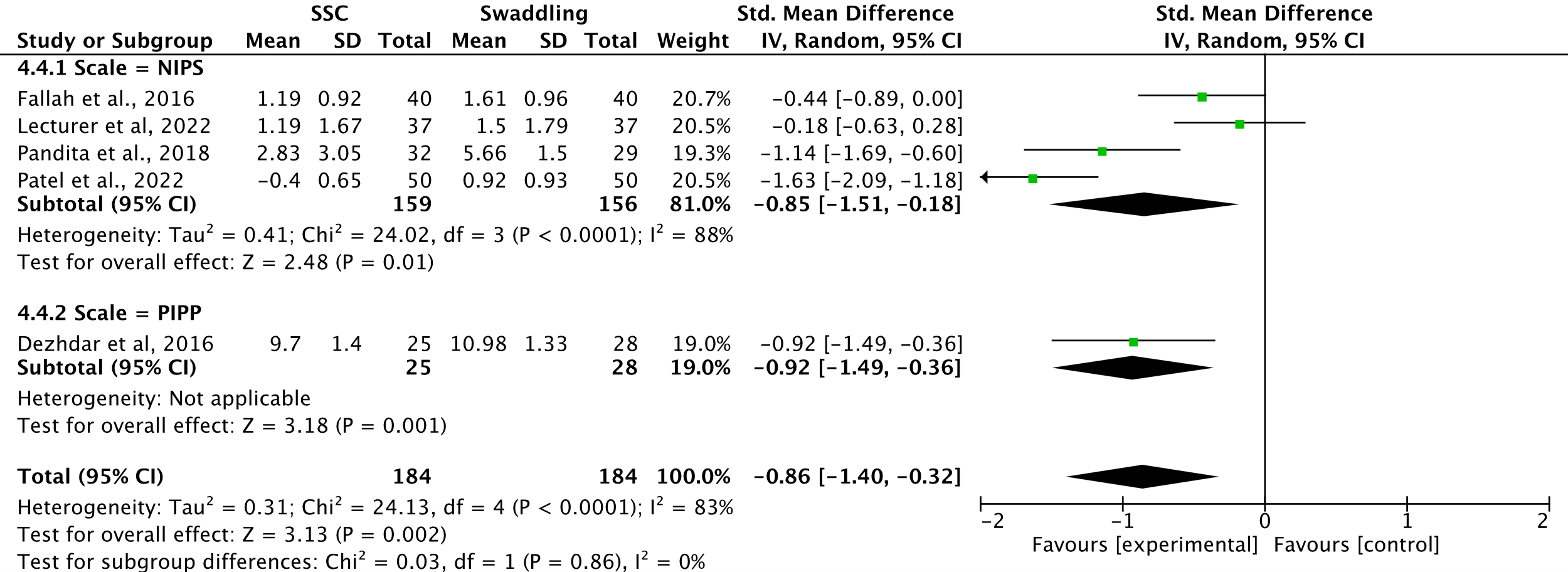


**Supplementary Figure 15** Meta-Analysis: Skin-to-Skin Contact vs. Swaddling for Procedural Pain in Newborns.


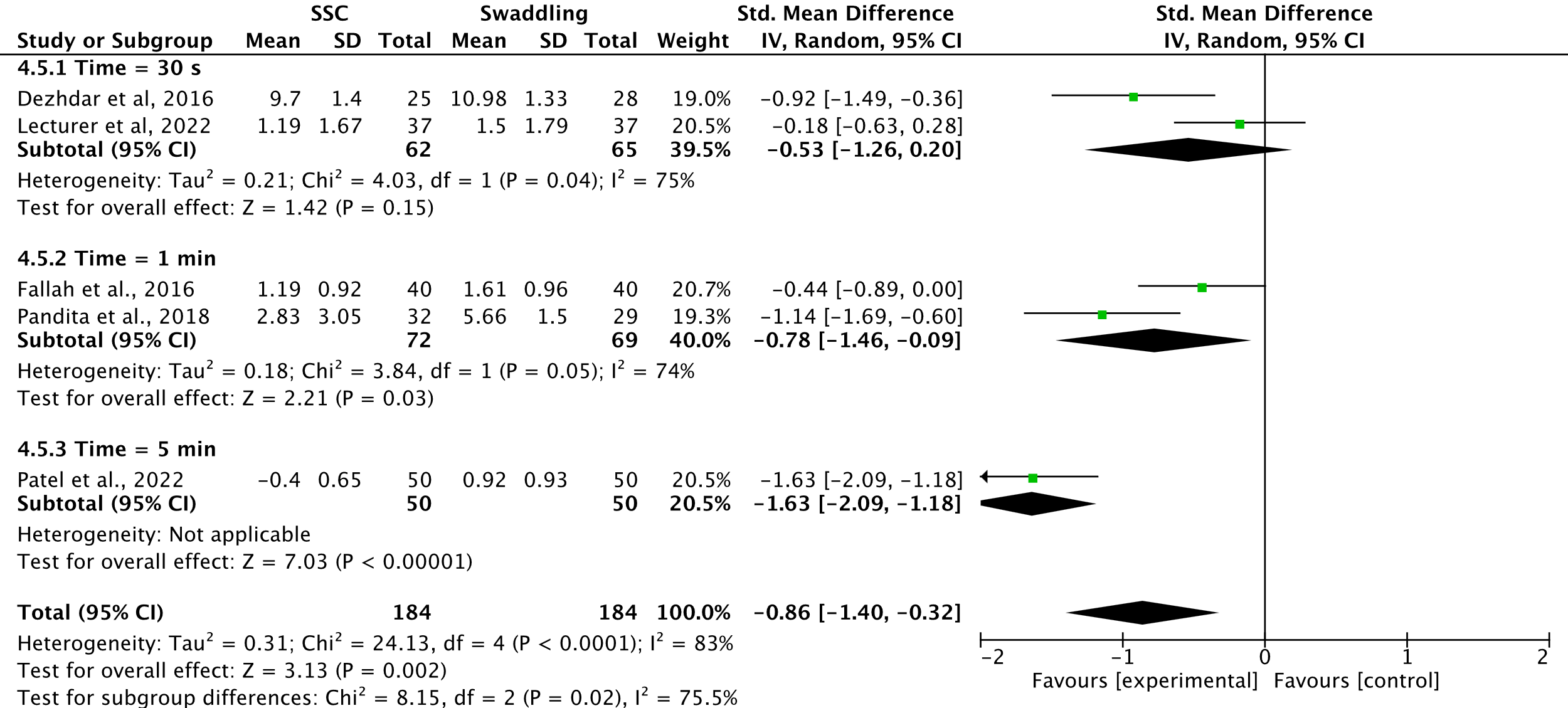


**Supplementary Figure 16** Meta-Analysis: Skin-to-Skin Contact vs. Swaddling for Procedural Pain in Newborns – Subgroup Analysis by Time of Measurement After the Procedure.
