# Supplementary material for: Impact of skin-to-skin contact on acute procedural pain in newborns: a systematic review and meta-analysis
Source: J Pediatr (Rio J). 2025 Sep 11;101(6):101442. doi: 10.1016/j.jped.2025.101442 (PMC12744622; doi:10.1016/j.jped.2025.101442)
Supplement: Supplementary file 4 [file mmc4.docx]

**JPED-D-25-00185_Supplementary Material**

| **Summary of Findings:** | | | | | | |
| --- | --- | --- | --- | --- | --- | --- |
| **Skin-to-skin contact compared to control in newborns undergoing painful procedures** | | | | | | |
| **Patient or population:** newborns undergoing painful procedures  **Context:**  **Intervention:** skin-to-skin contact  **Comparison:** control | | | | | | |
| Outcome Number of participants (studies) | Relative effect (95% CI) | **Potential absolute effects (95% CI)** | | | Certainty | What happens |
|  |  | **Control** | **Skin-to-skin contact** | **Difference** |  |  |
| Neonatal pain during clinical procedures assessed with: Neonatal pain was assessed using validated scales Number of participants: 1602 (19 RCTs) | - | - | - | SMD **1.14 lower SD** (1.6 lower to 0.68 lower) | ⨁⨁◯◯ Low^a,b,c^ | The evidence suggests that skin-to-skin contact results in a substantial reduction in neonatal pain during clinical procedures. |
| * **The risk in the intervention group** (and its 95% confidence interval) is based on the risk assumed from the comparator group and the **relative effect** of the intervention (and its 95% CI).  **CI:** Confidence interval; **SMD:** Standardized mean difference | | | | | | |
| **GRADE Working Group grades of evidence** **High certainty:** we are very confident that the true effect lies close to that of the estimate of the effect. **Moderate certainty:** we are moderately confident in the effect estimate: the true effect is likely to be close to the estimate of the effect, but there is a possibility that it is substantially different. **Low certainty:** our confidence in the effect estimate is limited: the true effect may be substantially different from the estimate of the effect. **Very low certainty:** we have very little confidence in the effect estimate: the true effect is likely to be substantially different from the estimate of effect. | | | | | | |

#### Explanations

^a.^ 42.1% of the studies were classified as high risk of bias in item 6 of RoB 2 for this outcome.

^b.^ High heterogeneity (I² = 92.7%), persistent even in subgroups.

^c.^ Wide confidence intervals in some subgroups and modest sample size in individual studies.

| **Summary of Findings:** | | | | | | |
| --- | --- | --- | --- | --- | --- | --- |
| **Skin-to-skin contact compared to carbohydrate solution in newborns undergoing painful procedures** | | | | | | |
| **Patient or population:** newborns undergoing painful procedures  **Context:** Skin-to-skin contact and the administration of carbohydrate solution are widely used non-pharmacological strategies for neonatal pain relief during clinical procedures. Both are low-cost, safe, and recommended in international guidelines. However, there are still uncertainties about which approach offers greater analgesic benefit, especially in preterm newborns and for diverse types of procedures. This comparison seeks to clarify the relative effectiveness of these interventions to guide evidence-based clinical practices.  **Intervention:** skin-to-skin contact  **Comparison:** carbohydrate solution | | | | | | |
| Outcome Number of participants (studies) | Relative effect (95% CI) | **Potential absolute effects (95% CI)** | | | Certainty | What happens |
|  |  | **Carbohydrate solution** | **Skin-to-skin contact** | **Difference** |  |  |
| Neonatal pain during clinical procedures assessed with: Neonatal pain was assessed using validated scales Number of participants: 919 (9 RCTs) | - | - | - | SMD **0.05 lower SD** (0.35 lower to 0.25 higher) | ⨁◯◯◯ Very low^a,b,c^ | The evidence is very uncertain about the effect of skin-to-skin contact on neonatal pain during clinical procedures. |
| * **The risk in the intervention group** (and its 95% confidence interval) is based on the risk assumed from the comparator group and the **relative effect** of the intervention (and its 95% CI).  **CI:** Confidence interval; **SMD:** Standardized mean difference | | | | | | |
| **GRADE Working Group grades of evidence** **High certainty:** we are very confident that the true effect lies close to that of the estimate of the effect. **Moderate certainty:** we are moderately confident in the effect estimate: the true effect is likely to be close to the estimate of the effect, but there is a possibility that it is substantially different. **Low certainty:** our confidence in the effect estimate is limited: the true effect may be substantially different from the estimate of the effect. **Very low certainty:** we have very little confidence in the effect estimate: the true effect is likely to be substantially different from the estimate of effect. | | | | | | |

#### Explanations

a. 56.6% of the studies were classified with high overall risk of bias (Item 6 for this outcome).

b. Considerable heterogeneity (I^2^ = 76.2%), with variation in the direction and magnitude of effects between studies.

c. Confidence interval crossing the line of no effect (SMD = –0.05; 95% CI: –0.35 to 0.25), with no statistical significance.

| **Summary of Findings:** | | | | | | |
| --- | --- | --- | --- | --- | --- | --- |
| **Skin-to-skin contact compared to breastfeeding in newborns undergoing painful procedures** | | | | | | |
| **Patient or population:** newborns undergoing painful procedures  **Context:** Skin-to-skin contact (KC) and breastfeeding (BF) are recommended non-pharmacological interventions for pain relief in newborns undergoing clinical procedures such as needle sticks, vaccinations, and injections. Both are safe, low-cost strategies that promote mother-infant bonding. However, few studies have directly compared these two approaches, and the available data remain limited. Evaluating their relative effectiveness is essential for guiding clinical protocols in neonatal units, prioritizing interventions with greater analgesic impact and better practical applicability.  **Intervention:** skin-to-skin contact  **Comparison:** Breastfeeding | | | | | | |
| Outcome Number of participants (studies) | Relative effect (95% CI) | **Potential absolute effects (95% CI)** | | | Certainty | What happens |
|  |  | **Breastfeeding** | **Skin-to-skin contact** | **Difference** |  |  |
| Neonatal pain during clinical procedures assessed with: Neonatal pain was assessed using validated scales Number of participants: 313 (4 RCTs) | - | - | - | SMD **0.44 higher SD** (0.21 higher to 0.66 higher) | ⨁⨁⨁◯ Moderate^a^ | Breastfeeding was significantly more effective than skin-to-skin contact in reducing neonatal pain during clinical procedures. |
| * **The risk in the intervention group** (and its 95% confidence interval) is based on the risk assumed from the comparator group and the **relative effect** of the intervention (and its 95% CI).  **CI:** Confidence interval; **SMD:** Standardized mean difference | | | | | | |
| **GRADE Working Group grades of evidence** **High certainty:** we are very confident that the true effect lies close to that of the estimate of the effect. **Moderate certainty:** we are moderately confident in the effect estimate: the true effect is likely to be close to the estimate of the effect, but there is a possibility that it is substantially different. **Low certainty:** our confidence in the effect estimate is limited: the true effect may be substantially different from the estimate of the effect. **Very low certainty:** we have very little confidence in the effect estimate: the true effect is likely to be substantially different from the estimate of effect. | | | | | | |

#### Explanations

a. 75% of the studies had a high overall risk of bias (Item 6 for this outcome).

| **Summary of Findings:** | | | | | | |
| --- | --- | --- | --- | --- | --- | --- |
| **Skin-to-skin contact compared to swaddling in newborns undergoing painful procedures** | | | | | | |
| **Patient or population:** newborns undergoing painful procedures  **Context:** Skin-to-skin contact and swaddling are non-pharmacological interventions used to reduce pain in newborns during clinical procedures such as needle sticks or vaccinations. Both promote comfort and self-regulation but work through different mechanisms: skin-to-skin contact involves physical closeness and emotional bonding, while swaddling provides physical containment and postural stability. Direct comparison between these interventions is still limited, making it essential to evaluate their relative effectiveness to guide neonatal healthcare professionals in choosing the most effective analgesic approach in clinical settings.  **Intervention:** skin-to-skin contact  **Comparison:** swaddling | | | | | | |
| Outcome Number of participants (studies) | Relative effect (95% CI) | **Potential absolute effects (95% CI)** | | | Certainty | What happens |
|  |  | **Swaddling** | **Skin-to-skin contact** | **Difference** |  |  |
| Neonatal pain during clinical procedures assessed with: Neonatal pain was assessed using validated scales Number of participants: 368 (5 RCTs) | - | - | - | SMD **0.86 lower SD** (1.38 lower to 0.34 lower) | ⨁⨁⨁◯ Moderate^a^ | Skin-to-skin contact likely reduces neonatal pain during clinical procedures. |
| * **The risk in the intervention group** (and its 95% confidence interval) is based on the risk assumed from the comparator group and the **relative effect** of the intervention (and its 95% CI).  **CI:** Confidence interval; **SMD:** Standardized mean difference | | | | | | |
| **GRADE Working Group grades of evidence** **High certainty:** we are very confident that the true effect lies close to that of the estimate of the effect. **Moderate certainty:** we are moderately confident in the effect estimate: the true effect is likely to be close to the estimate of the effect, but there is a possibility that it is substantially different. **Low certainty:** our confidence in the effect estimate is limited: the true effect may be substantially different from the estimate of the effect. **Very low certainty:** we have very little confidence in the effect estimate: the true effect is likely to be substantially different from the estimate of effect. | | | | | | |

#### Explanations

a. 60% of the studies showed a high risk of bias (Item 6 for this outcome).
